# Dietary fibers boost gut microbiota-produced B vitamin pool and alter host immune landscape

**DOI:** 10.1186/s40168-024-01898-7

**Published:** 2024-09-23

**Authors:** Erica T. Grant, Amy Parrish, Marie Boudaud, Oliver Hunewald, Akiyoshi Hirayama, Markus Ollert, Shinji Fukuda, Mahesh S. Desai

**Affiliations:** 1 Department of Infection and Immunity, Luxembourg Institute of Health, 4354 Esch-Sur-Alzette, Luxembourg; 2 Faculty of Science, Technology and Medicine, University of Luxembourg, 4365 Esch-Sur-Alzette, Luxembourg; 3 Institute for Advanced Biosciences, Keio University, Yamagata, 997-0052 Japan; 4 Transborder Medical Research Center, University of Tsukuba, Ibaraki, 305-8575 Japan; 5 Gut Environmental Design Group, Kanagawa Institute of Industrial Science and Technology, Kanagawa, 210-0821 Japan; 6 Odense Research Center for Anaphylaxis, Department of Dermatology and Allergy Center, Odense University Hospital, University of Southern Denmark, 5000 Odense, Denmark

**Keywords:** Microbiome, Dietary fiber, B vitamins, Mass cytometry

## Abstract

**Background:**

Dietary fibers can alter microbial metabolic output in support of healthy immune function; however, the impact of distinct fiber sources and immunomodulatory effects beyond short-chain fatty acid production are underexplored. In an effort to discern the effects of diverse fibers on host immunity, we employed five distinct rodent diets with varying fiber content and source in specific-pathogen-free, gnotobiotic (containing a 14-member synthetic human gut microbiota), and germ-free mice.

**Results:**

Broad-scale metabolomics analysis of cecal contents revealed that fiber deprivation consistently reduced the concentrations of microbiota-produced B vitamins. This phenomenon was not always explained by reduced biosynthesis, rather, metatranscriptomic analyses pointed toward increased microbial usage of certain B vitamins under fiber-free conditions, ultimately resulting in a net reduction of host-available B vitamins. Broad immunophenotyping indicated that the local gut effector immune populations and activated T cells accumulate in a microbiota-dependent manner. Supplementation with the prebiotic inulin recovered the availability of microbially produced B vitamins and restored immune homeostasis.

**Conclusions:**

Our findings highlight the potential to use defined fiber polysaccharides to boost microbiota-derived B vitamin availability in an animal model and to regulate local innate and adaptive immune populations of the host.

Video abstract.

**Supplementary Information:**

The online version contains supplementary material available at 10.1186/s40168-024-01898-7.

## Background

The discovery of novel ways to characterize the composition, functional capacity, and metabolic activity of the microbiome has led to an explosion of linkages between the activity of this complex super organ and host health [1]. Meanwhile, as immune disorders and inflammatory diseases become more prevalent, there is a growing need to develop a mechanistic understanding of microbiome–host crosstalk in order to leverage microbiome-based therapies in disease treatment [2]. Diet is a major determinant of microbial metabolic output [2–4] and therefore a promising tool to improve existing treatment strategies, provided that concrete links can be made between dietary components, microbial changes, and the subsequent host immune phenotype.

In particular, dietary fiber has strong effects on the microbiota composition and metabolic output [5]. Fermentation of dietary fiber by gut bacteria occurs in the lower gastrointestinal (GI) tract—cecum and colon in mice or large intestine in humans—yielding short-chain fatty acid (SCFAs) that can upregulate regulatory T cells (Treg) [6, 7]. However, the structures of dietary fibers can vary considerably and, thus, the bacteria possessing the enzymatic capacity to cleave specific glycosidic linkages are similarly diverse. Little is known about how specific dietary fibers alter bacterial metabolic output to impact the host immunophenotype at a broader level. In a high-fiber dietary intervention among 18 healthy adults, Wastyk et al. documented increases in the overall carbohydrate-degrading capacity of participants’ gut microbes and individualized broad-scale immune responses [8]. Surprisingly, fecal SCFA concentrations were diminished on the high-fiber diet, underscoring the need to consider the role of other relevant microbial byproducts that can regulate host immunity [9], including microbially produced vitamins [10], and secondary bile acids [11].

Based on publicly available metagenomic data, previous studies have detailed B vitamin biosynthesis pathways in the gut microbiomes of diverse mammalian hosts [12], including humans [13, 14]. Loss of microbial genes involved in B vitamin production has been shown to be associated with disease among patients with inflammatory bowel diseases (IBD) [15] and type 2 diabetes [13]. B vitamins are critical cofactors for various host and bacterial metabolic and immunoregulatory processes. With the majority of dietary B vitamins absorbed in the small intestine [16, 17], an intricate ecological balance exists in the lower GI tract between subsets of gut bacteria that are able to synthesize B vitamins and those that lack these critical synthesis pathways, instead expressing transporters to sequester these vital cofactors [14]. Consequently, dynamics of the synthesis and utilization of B vitamins by gut bacteria have a direct impact on host availability to uptake these critical cofactors. Antioxidative vitamins, such as riboflavin (vitamin B2), can help maintain anaerobic conditions in the colon, allowing proliferation of the anti-inflammatory gut commensal *Faecalibacterium prausnitzii* [18]. Despite the fact that gut bacteria have long been known to synthesize B vitamins in vitro [19], and germ-free rodents are known to have higher requirements of dietary B vitamins [20, 21], whether defined dietary changes affect B vitamin synthesis by specific gut microbes and the mechanisms underlying these alterations are not known.

To better understand how fibers alter microbial metabolism and host immunity, we employed two distinct commercial and three customized rodent diets with varying sources and content of dietary fiber in mice with both native and humanized gut microbiomes (Supplementary Table 1). Broad immunophenotyping of 128 samples using time-of-flight mass cytometry (CyTOF) of host local (colonic lamina propria, *n* = 68) and systemic organs (spleen, *n* = 44; lung, *n* = 16) revealed a variety of immune biomarkers which are differentially affected by fiber sources and quantity. Moreover, using broad-scale capillary electrophoresis time-of-flight mass spectrometry-based (CE-TOFMS) microbial metabolomics, we identified a suite of microbial metabolites such as secondary bile acids and B vitamins whose abundance is influenced by the microbial fermentation of dietary fiber. Microbial metatranscriptomic analyses helped to identify subsets of gut bacteria that synthesize or utilize B vitamins, with deprivation of dietary fiber resulting in increased downstream use of microbial B vitamins and consequently local availability to the host. Our study creates a link between the microbial fermentation of dietary fiber, microbial production of B vitamins, and their possible effects on the immune system. Future studies can make use of these findings to further explore the potential health implications.

## Methods

### Mice

All experiments were performed according to the “Règlement grand-ducal du 11 janvier 2013 relatif à la protection des animaux utilisés à des fins scientifiques” based on the “Directive 2010/63/EU” on the protection of animals used for scientific purposes. Experiments were performed with 6–8-week-old female BALB/c mice (Charles River Laboratories, France) housed either in the specific-pathogen-free (SPF) facility of the Luxembourg Institute of Health or in the germ-free facility of the University of Luxembourg. Germ-free BALB/c mice were bred in isolators and transferred to iso-cages prior to experimental feeding and/or 14SM colonization. Gnotobiotic work was approved by the Luxembourgish Ministry of Agriculture, Viticulture and Rural Development (LUPA 2019/50). Mice were fed on their respective diets for 40 days and then sacrificed by cervical dislocation.

### Diet formulation

Compositions of all diets used in this study can be found in Supplementary Table 1. SPF SC1 mice were fed the Rat and Mouse Breeder and Grower diet (Special Diets Services, catalogue no. 801722); gnotobiotic SC1 mice were fed the germ-free equivalent standard rodent chow (SAFE®R04, Augy, France) with fiber sourced from cereals. Although the SPF and gnotobiotic diets originate from different suppliers, overall compositions were highly comparable and are not expected to present unique glycan linkages, thus both are referred to as SC1 in the text. All mice were maintained on the SC1 diet before the start of the experiment (D0). SC2 mice were fed the Autoclavable Rodent Breeder diet (LabDiet, catalogue no. 5013) with fiber from cereals, alfalfa, yeast, and beet. The fiber-free (FF) diet was formulated based on Desai et al. [22] and is a modified version of Harlan TD.08810 diet [23], with glucose replacing the starch and maltodextrin components (SAFE, Augy, France). The inulin (IN)- and fiber-supplemented (FS) diets are based on the FF diet, with 10% of the dextrose replaced by either purified inulin derived from chicory root (Acros Organics, catalogue no. AC457105000, Lot no. A0404347) or a mix of equal parts crude raw fibers from apple, wheat, oat, pea, and psyllium (J. Rettenmaier & Sohne, Germany).

### Bacterial culturing and colonization of 14-member synthetic microbiota

Culturing of the 14-member synthetic human microbiota (14SM) and colonization in germ-free mice was performed according to Steimle et al. [24], which was adapted from a previous work [22]. The 14SM community is composed of the following bacteria (type strain unless otherwise indicated): *Akkermansia muciniphila*, *Bacteroides caccae*, *Bacteroides ovatus*, *Bacteroides thetaiotaomicron*, *Bacteroides uniformis*, *Barnesiella intestinihominis*, *Clostridium symbiosum*, *Collinsella aerofaciens*, *Desulfovibrio piger*, *Escherichia coli* HS, *Eubacterium rectale* A1-86, *Faecalibacterium prausnitzii* A2-165, *Marvinbryantia formatexigens*, and *Roseburia intestinalis*. Bacterial colonization was verified by qPCR from DNA extracted from fecal samples 7 days after oral gavage.

### Organ and tissue processing

Spleens and lungs were excised and placed in ice-cold 1 × PBS. Approximately 1–2 cm of proximal colon and distal ileum were placed in 1 mL RNAprotect Tissue Reagent, and the remaining tissues were placed on ice in 5 mL Hank’s balanced salt solution (HBSS) without Ca2^+^ and Mg2^+^ containing 10 mM HEPES. Colonic lamina propria cells were extracted using the Lamina Propria Dissociation Kit (Miltenyi Biotec, catalogue no. 130–097-410) and gentleMACS Dissociator (Miltenyi Biotec, catalogue no. 130–093-235) according to the manufacturer’s instructions. Splenocytes were isolated by manually dissociating tissue over a 70-μm filter and washing with ice-cold 1 × PBS. The samples were washed and re-suspended in FACS buffer until subsequent cell staining. Lung tissues were transferred into a petri dish and cut into small fragments with a scalpel. The tissue fragments were digested for 1 h at 37 °C in a digestion buffer consisting of 100 μL collagenase II, 750 μL fetal bovine serum (FBS), 1.5 μL benzonase, 7.5 μL MgCl2, and 7.5 mL 1 × PBS. Solutions were then poured over a 40-μm cell strainer (Falcon), and manual digestion was performed with a 10-mL syringe plunger. The filter was washed with Dulbecco’s modified Eagle medium (DMEM) and centrifuged at 1500 rpm for 5 min. Cell pellets were re-suspended in ACK lysing buffer and incubated for 1 min at room temperature. Next, 10 mL of DMEM was added and cells were washed twice by centrifugation at 1500 rpm for 5 min. After digestion, cells were re-suspended in a buffer composed of 1 × PBS and 0.5% bovine serum albumin (BSA), pH 7.2.

### Mass cytometry

Cell staining of fresh single cells was performed according to Wolter et al. [25]. Briefly, a total of 3.0 × 10^6^ cells per organ per animal were transferred into 15-mL conical tubes. For live/dead staining, cells were incubated with 5 μM cisplatin for 5 min. Cells were washed, and cell surface staining mix (Supplementary Table 3) was added containing pre-conjugated antibodies for 30 min at room temperature. Samples were washed twice with FACS buffer, then fixed using the FoxP3 Fix/Perm kit (eBiosciences) for 45 min at 4 °C, followed by permeabilization wash. Samples were then incubated with the intracellular staining mix for 30 min at room temperature. Cells were washed with FACS buffer twice, and pellets were resuspended in Cell-ID™ Intercalator-Ir (Fluidigm) in MaxPar fixation solution (Fluidigm, catalogue no. 201192B) and refrigerated overnight, or for up to 5 days. Prior to acquisition, samples were washed twice with 1 × PBS, and then washed twice with deionized water. Cell pellets were further resuspended in deionized water at 0.5 × 10^6^ cells/mL and topped up with 10% calibration beads (EQ Four Element Calibration Beads, Fluidigm). All samples were acquired on the Helios Mass Cytometer (Fluidigm).

### Mass cytometry data analysis

Each organ was analyzed independently following the same analysis pipeline, as previously described by Leonard et al. [26]. FCS files were normalized with the normalization passport EQ-P13H2303_ver2. FCS files were uploaded into FlowJo™ software [27] for cleaning. First, beads were removed, followed by DNA^−^negative events, doublets, and dead cells. CD45^+^ cells were exported into new FCS files. Unsupervised analysis was carried out by importing the files into RStudio version 1.0.143 (R version 3.4.4) using the R package “flowcore” version 1.44.2 [28] and FlowSOM version 2.6.0 [29]. All selected lineage and functional markers underwent arcsinh transformation with a cofactor of 5. Single cells were clustered using FlowSOM [29] into 100 som-clusters (grid-size 10 × 10, 10 epochs). Without further meta-clustering, those som-clusters were visualized in a heatmap of median marker intensity (with each marker transformed between 0 and 1) (Supplementary Fig. 1 for cLP). Clusters were then annotated into phenotypes by two independent researchers. Similar populations of biological relevance were manually merged. Counts per cluster were normalized based on sample size. Manual gating of CD4^+^ populations was performed in FlowJo (TriStar). CyTOF datasets showing immune cell populations in cLP, spleen, and lung expressed as a percentage of all CD45^+^ cells can be found in Supplementary Table 4–6. Note that the 14SM (*n* = 12) cLP data were previously analyzed using manual gating in FlowJo [25]; these files were re-analyzed in this study using the unsupervised clustering pipeline for comparison to SPF samples.

### 16S rRNA gene sequencing and analysis

Fecal pellets were stored at –20 °C until DNA extraction using phenol–chloroform, according to Steimle et al. [24]. Final DNA concentration was determined with Qubit® dsDNA HS assay kit on a Qubit® 3.0 fluorometer (Life Technologies, Eugene, Oregon, USA). Sequencing of the V4 region of the 16S rRNA gene was performed on an Illumina MiSeq system, as described by Neumann et al. [30]. Raw sequences were processed using QIIME2 version 2020.6 [31] with DADA2 for sequence quality control and taxonomic assignment was performed using VSEARCH against the SILVA 138 reference database [32] (Supplementary Table 2). Alpha diversity was calculated using the core diversity metrics tool in QIIME2. Further analyses were performed in R version 4.0.2 [33] using the package “phyloseq” version 1.34.0 [34]. Taxa not observed more than once on average across all samples were removed and the data was rarefied to the minimum library size. PCoA plots were generated using the package “vegan” version 2.5–7 [35], with clustering significance testing using package “pairwiseAdonis” version 0.4 [36]. Differential enrichment analysis was performed using the package “DESeq2” version 1.30.1 [37]. Visualizations were generated using “ggplot2” version 3.3.5 [38] and “forcats” version 0.5.1 [39]. Note that the SC1 and FF mice were included in a subsequent experiment; therefore, the 16S rRNA gene sequencing data at D0 and D40 also appear in a separate study by Parrish et al. [40]. Likewise, the microbiome compositions of abundances of the 14SM mice were previously confirmed using strain-specific primers by qPCR [25].

### Short-chain fatty acid quantification

SCFA analysis was performed on flash-frozen cecal content stored at –80 °C. Approximately 50 mg of the sample was mixed with 500 μL MilliQ water with 2-ethylbutyric acid. Samples were homogenized with 5 ceramic beads (1.4 mm) using a Precellys24 homogenizer (Bertin Technologies) at 6000 rpm 1 × 30 s. Samples were then centrifuged at 21,000 × *g* for 5 min at 4 °C and analyzed by GC–MS according to Greenhalgh et al. [41].

### CE-TOFMS-based metabolomics

Cecal and serum metabolites were processed and analyzed on an Agilent CE system, as described by Steimle et al. [42].. Metabolite concentrations can be found in Supplementary Tables 7–9. For data analysis, metabolites which were present in at least half of one experimental group were included. Metabolite concentrations were normalized to the cecal weight or serum volume and log-transformed, then scaled for each metabolite by mean-centering and dividing by standard deviation. Heatmaps of top differentially abundant cecal metabolites (i.e., lowest *P* values) were generated based on a linear mixed-effect model using limma with the FF group as reference [43] in MetaboAnalyst 5.0 [44]. Visualization of intersecting sets was performed using “ComplexUpset” version 1.3.3 [45, 46] and “ggVennDiagram” version 1.2.0 [47]. PCA plots were generated using the “stats” version 4.0.2 [33], “factoextra” version 1.0.7 [48], and members of the tidyverse [39] ecosystem (“dplyr” version 1.0.5 [49], “ggfortify” version 0.4.13 [50], “ggforce” version 0.3.3 [51], “ggrepel” version 0.9.1 [52]). Correlation matrix between B vitamin concentrations and immune cell frequencies was calculated using “corrplot” version 0.92 [53].

### RNA extraction

Cecal contents were flash-frozen in liquid nitrogen and stored at –80 °C until RNA extraction. Cecal contents were maintained on dry ice while 1 mL RNAProtect Bacteria Reagent was added. Samples were thawed on wet ice for 10 min, followed by centrifugation and removal of RNAProtect Bacteria Reagent. Approximately 250 µl acid-washed glass beads were added to each sample, along with 500 µl of Buffer A, 210 µl of 20% SDS, and 500 µl of phenol:chloroform:isoamyl alcohol (25:24:1) pH 4.3. Samples were homogenized on a RETSCH Mixer Mill MM 400 for 5 min at 30 Hz, then centrifuged at 4 °C for 3 min at 13,000 rpm. The supernatant was transferred to a new tube and 500 µl of phenol:chloroform:isoamyl alcohol (25:24:1) pH 4.3 was added to each sample. Samples were again centrifuged at 4 °C for 3 min at 13,000 rpm and the aqueous phase recovered. A one-tenth volume of 3 M sodium acetate (pH 5.5) and 1 volume of –20 °C 100% ethanol were added and then mixed by gentle inversion. Samples were incubated for 20 min on wet ice then centrifuged at 4 °C for 20 min at 13,000 rpm. The pellet was recovered and washed in 500 µl of cold 70% ethanol, then centrifuged at 4 °C for 5 min at 13,000 rpm. The ethanol wash step was repeated once more before air drying the pellet for 10 min and re-suspending in 50 µl nuclease-free water. Genomic DNA was digested DNAse I for 30 min at 37 °C, then 1 µl of 0.5 M EDTA was added and the DNAse I was heat-inactivated for 10 min at 65 °C. The RNA was purified using the RNeasy mini kit (Qiagen) according to manufacturer’s instructions and eluted RNA was stored at –80 °C. Prior to sequencing, RNA quality was assessed using a 2100 Bioanalyzer RNA Nanochip (Agilent, Santa Clara, CA).

### RNA sequencing and analysis

RNA libraries were prepared using the Illumina® Stranded Total RNA Prep with Ribo-Zero Plus kit to remove rRNAs from mouse and bacterial sources. Samples were sequenced in a 2 × 75 bp arrangement, at a depth of 40 M reads per sample using NovaSeq 6000 SP Reagent Kit v1.5 (Illumina, San Diego, CA, USA) on an Illumina NovaSeq 6000 system. Fastq files were processed using KneadData, within the bioBakery suite of workflows [54]. Adapters were removed using Trimmomatic [55] and fragments below 50% of the total expected read length were filtered out. Bowtie2 [56] was used to map and remove contaminant reads corresponding to either rRNA databases or the *Mus musculus* genome. Clean fastq files were concatenated before passing to HUMAnN 3 for metagenome mapping with MetaPhlAn 3 [57]. In this pipeline, unaligned reads were translated using DIAMOND [58] for protein identification. Gene family abundances, pathway abundances, and pathway coverage data for all samples were joined into a single table and re-calculated as counts per million reads (CPM) and relative abundance. Enzymes mapping to B vitamin synthesis, downstream use, or transport were manually annotated based on the relevant PubSEED Subsystems, as described in Magnúsdóttir et al. [14] with results stratified by contributing bacteria (Supplementary Table 10). The classification of transcripts corresponding to enzymes up- or downstream of vitamin synthesis was performed considering the form that is absorbed in the colon. In the case of vitamin B1, while the free dietary form is taken up through passive diffusion or active transport before being phosphorylated by host enzymes in the small intestine, in the colon, the biologically active form, thiamine pyrophosphate (TPP), is absorbed as TPP through the colonic TPP transporter [59]; therefore, we classify the enzyme responsible for this conversion as upstream. Similarly, while the host passively takes up the dephosphorylated forms of pantothenic acid (vitamin B5, measured as pantothenate) and pyridoxine, pyridoxal, and pyridoxamine (vitamin B6) [10], we note that endogenous host enzymes can readily dephosphorylate this compound in the lumen; therefore, we still consider the bacterial kinases to be upstream of vitamin production. In any case, we note that the abundance of transcripts mapping to these particular enzymes are overall low and consistent between diets; therefore, their classification does not alter the overall conclusions. UniRef IDs were mapped to enzyme classification (EC) identifiers, stratified by contributing taxon (Supplementary Table 11), to generate the plots of bacterial enzymes in closely involved in B vitamin metabolism (Fig. 6c, Supplementary Figs. 6, 7).

### Statistical analyses

Unless otherwise stated, outliers were detected and excluded using the ROUT method (*Q* = 1%). Cleaned data was checked for normality according to the Shapiro–Wilk test. If all groups were normally distributed, a Brown–Forsythe and Welch ANOVA test (no assumption of equal variance) was used; otherwise, a Kruskal–Wallis test was used. Adjustment for multiple comparisons was performed using the Benjamini–Hochberg method using GraphPad Prism version 9.0.0 for Windows (GraphPad Software, San Diego, California USA, www.graphpad.com).

## Results

### Dietary fiber deprivation broadly changes local immune landscape via the gut microbiota

**Fig. 1:**
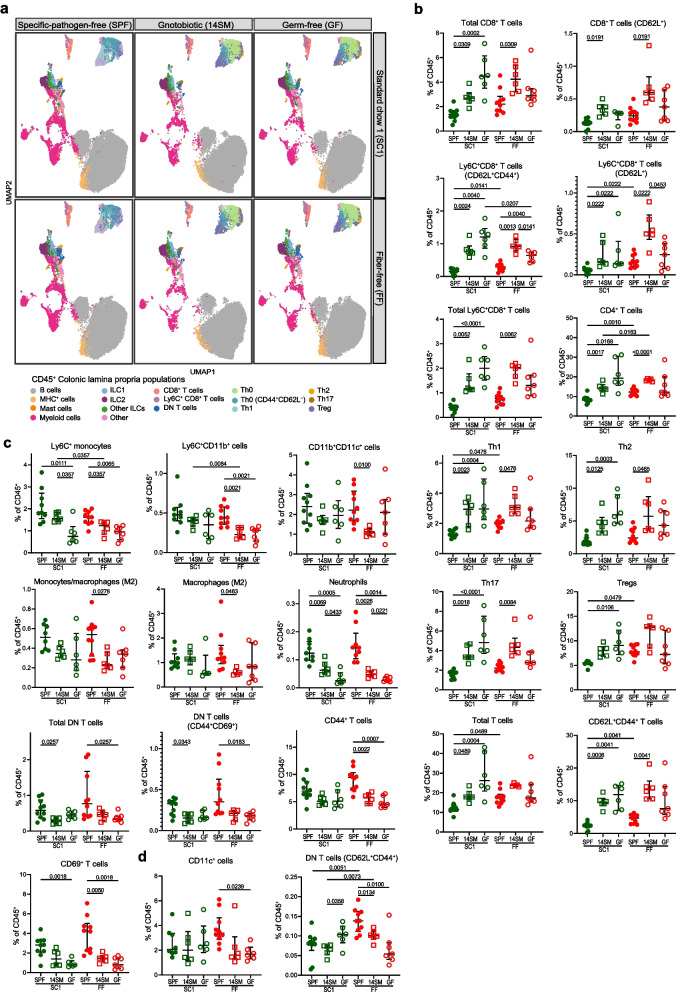
Colonization state exerts stronger impact on immune profiles than diet in CyTOF analysis. **a** UMAP projections of the cLP populations in SC1 and FF-fed mice, faceted by colonization state (specific-pathogen-free, SPF; 14-member synthetic microbiota, 14SM; germfree, GF). Plots were generated from 100 clusters with populations manually annotated and merged. Dot plots show immune clusters of the colonic lamina propria (cLP) with proportions among SC1 (green) and FF (red) groups for the three colonization states with either **b** negative or **c** positive association between microbial colonization and population abundance as % of total CD45^+^ cells. **d** Variance dominated by interaction between diet and colonization state. CD4^+^ populations were manually gated in FlowJo. Brown–Forsythe and Welch ANOVA or Kruskal–Wallis test with *P* values adjusted using the Benjamini–Hochberg method. *n* = 6–10 mice per group (SPF: two batches of five mice each; 14SM: two batches of three mice each; GF: two batches of two to four mice each). Outliers were removed based on ROUT (*Q* = 1%): Tregs SPF SC1 *n* = 1; CD11c^+^ cells SPF SC1 *n* = 2 and GF FF *n* = 1; CD11b^+^CD11c^+^ cells 14SM FF *n* = 1; Macrophages (M2) SPF SC1 *n* = 1 and 14SM FF *n* = 1; monocytes/macrophages (M2) SPF SC1 *n* = 2; neutrophils SPF SC1 *n* = 1 and SPF FF *n* = 1. Specific-pathogen-free (SPF) = closed circles, gnotobiotic (14SM) = open squares, germ-free (GF) = open circles

**Fig. 2:**
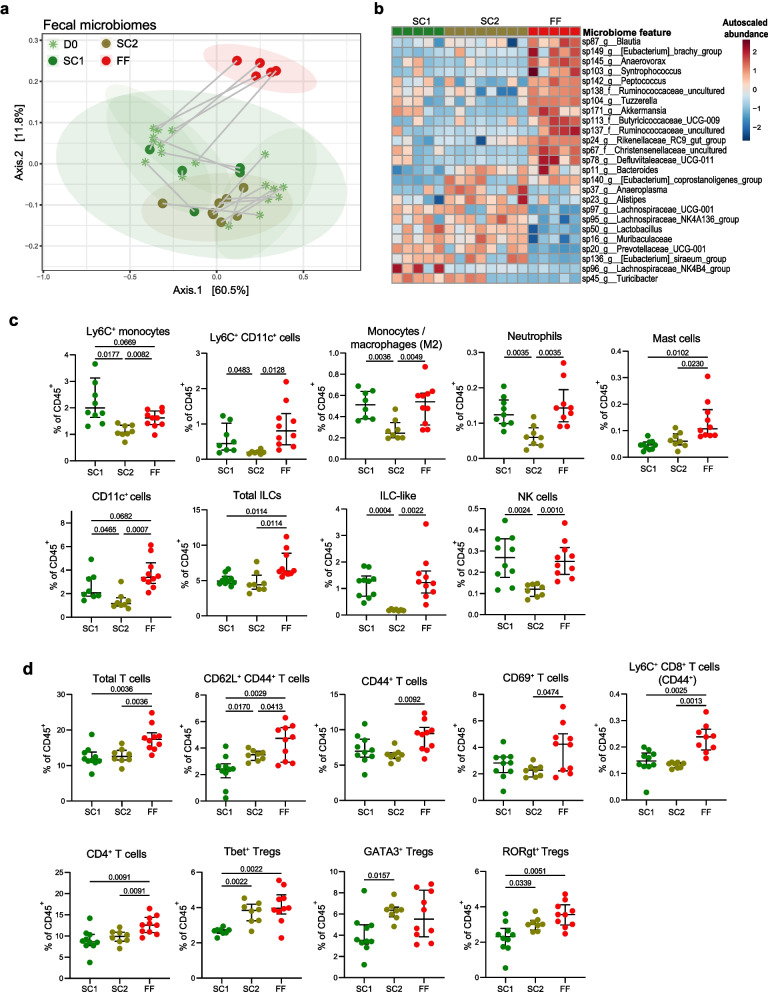
Fiber deprivation shifts microbiome composition and local immunity in mice with complex microbiome. **a** Principal coordinates of analysis (PCoA) based on Bray–Curtis dissimilarity index from fecal microbiota of SPF mice at day 0 (D0, stars, light green), when all mice were fed standard chow 1 (SC1), and after feeding for 40 days (D40, dots) on either SC1, standard chow 2 (SC2), or fiber-free (FF) diet (green, brown, and red, respectively). Ellipses show 95% confidence interval (CI). **b** Heatmap of top 25 differentially abundant taxa in mice fed SC1, SC2, or FF diets according to a linear mixed-effect model with the FF group as reference. Microbiome feature abundances were normalized by total sum scaling and log-transformed, then auto-scaled for each taxa by mean-centering and dividing by standard deviation. Feature order is based on Ward clustering; sample order is fixed by group. For **a** and **b**, *n* = 5 (SC1 and FF) or 8 (SC2) mice per group. Adaptive and innate immune clusters of the cLP, represented as % of total CD45^+^ cells, showing significantly different proportions among **c** innate and **d** adaptive populations. CD4^+^ T cell populations were manually gated in FlowJo. Outliers were removed based on ROUT (*Q* = 1%): Tbet^+^ Tregs SC1 *n* = 2; Ly6C^+^ Monocytes SC1 *n* = 1; Ly6C^+^ CD11c^+^ cells SC1 *n* = 2 and SC2 *n* = 1; M2 Mono/Macro SC1 *n* = 2. Statistical testing was performed using Brown–Forsythe and Welch ANOVA or Kruskal–Wallis test with *P* values adjusted using the Benjamini–Hochberg method. For **c**–**d**, *n* = 10 (SC1 and FF; two batches of five mice each) or 8 (SC2; four batches of four mice each) mice per group. Only populations with significant differences between the diets are shown; see Supplementary Table 3 for a complete list of populations analyzed

Deprivation of dietary fiber has been reported to negatively affect colonic Tregs through the decreased microbially produced SCFA butyrate [6, 7]. However, it is less clear how fiber deprivation impacts other immune cell populations via the gut microbiota. To assess the impact of the fiber-free (FF) diet (Supplementary Table 1) on local and systemic immunity, broad immunophenotyping was performed by time-of-flight mass cytometry (CyTOF) with a 28-marker panel (Supplementary Table 2) on the colonic lamina propria (cLP) (Figs. 1 and 2, Supplementary Fig. 1, Supplementary Table 3), spleen (Supplementary Fig. 2a–c, Supplementary Table 4), and lung (Supplementary Fig. 3a–c, Supplementary Table 5). We simultaneously used germ-free (GF) and gnotobiotic BALB/c control mice colonized with a 14-member synthetic human microbiota (14SM) [22] to control for diet-specific effects in the absence of a microbiome and to assess the ability of the 14SM community to recapitulate an SPF immune phenotype [22] (Fig. 1).

The unique immunophenotype signature of the fiber deprivation (SC1 vs FF) and the three colonization states (SPF, 14SM, or GF) was illustrated with Uniform Manifold Approximation and Projection (UMAP) [60] for dimension reduction (Fig. 1a). The SPF profiles of both diets were characterized by a reduction of Th0 cells (replaced by Th0 expressing CD44^+^ and CD62L^+^). Populations that were reduced in the presence of a gut microbiota included CD8^+^ T cells, mostly driven by increases in the CD62L^+^, CD62L^+^CD44^+^, Ly6C^+^CD62L^+^, and Ly6C^+^ subpopulations (Fig. 1b). These markers reflect a central memory phenotype that is reasonable given the low local stimulation the gut of a GF mouse, but less expected in the gnotobiotic (14SM) condition, which appears to present an intermediate phenotype between GF and SPF. It is possible that the simplified community presents less diverse antigens, including lack of microbial members that were not analyzed in this study, such as viruses or fungi, which may contribute to a more muted local immune response relative to SPF mice. There was also a negative trend with colonization and proportion of CD4 + T cells and Th subtypes, including Tregs. These differences may be explained by a general elevation in the proportions of CD3^+^ cells, especially those expressing CD62L^+^CD44^+^, which again suggests a bias toward central memory cell types and homing away from the colon. SPF mice on both diets exhibit elevated proportions of local tissue resident (CD44^+^) and activated T cell types (CD69^+^), especially due to an increase in double negative (DN) T cells (Fig. 1c). This population is likely comprised of mucosa-associated invariant T (MAIT) cells, invariant natural killer T, and TCRγδ + T cells, the former of which recognizes microbial B vitamin metabolites [61]. Innate myeloid cell subsets including neutrophils, CD11b^+^CD11c^+^ cells, Ly6C^+^CD11b^+^ cells, LyC6^+^ monocytes, and M2 monocytes and macrophages were also positively associated with colonization (Fig. 1c). Altogether, these data obtained from SPF, gnotobiotic, and germ-free models identify a microbiota-dependent local immune dysregulation involving broader types of immune cell populations. While colonization state generally played a stronger role in shaping immune profiles compared to diet, for two clusters, identified as CD11c^+^ cells and DN T cells expressing CD62L^+^ and CD44^+^, the highest source of variation was attributed to the interaction of diet and colonization status (Fig. 1d).

Diet and colonization had a predictably strong impact on local immune populations; however, we also noted immune populations which were significantly affected by diet among spleen (Supplementary Fig. 2c) and lung (Supplementary Fig. 3c) immune cell profiles. With the exception of one cell cluster annotated as Ly6C^+^CD8^+^ T cells (CD62L^+^CD44^+^) in the cLP, no differential changes were observed in germ-free cLP, spleen, or lung cell populations based on diet, highlighting the essential role of the microbiome to have an effect locally (Fig. 1) and peripherally (Supplementary Figs. 2 and 3).

### Standard lab chows differentially modulate gut microbiota and local immunity

To better understand broader level changes in the immune system, we fed SPF mice for 40 days on an FF diet [22] (two independent experiments of five mice each). We used two standard laboratory rodent chow diets from independent manufacturers with similar crude fiber percentages (SC1 and standard chow 2 (SC2), as high fiber contrasts to the FF diet. SC2 is composed of more diverse fiber sources, including cereals, alfalfa, yeast, and beetroot, compared to only cereals in the SC1 diet (Supplementary Table 1).

In SPF mice, 16S rRNA gene sequencing of fecal samples (Supplementary Table 6) revealed a significant shift in the microbiota compositions between the two standard chows and the custom fiber-free diets (SC1 vs FF, *P* = 0.005; SC2 vs FF, *P* = 0.001) after 40 days of feeding, as illustrated in the principal coordinates of analysis (PCoA) plot using Bray–Curtis dissimilarity distances (Fig. 2a). The clustering of SC1 and SC2 fed mice did not significantly differ at D40 (*P* = 0.119). All mice were fed the SC1 diet for at least 1 week before being shifted to a new diet on D0; therefore, shifts from D0 to D40 were expected for all groups except SC1. Nonetheless, we still observed a slight shift in the clusters between day 0 and day 40 under the SC1 diet (*P* = 0.042), likely owing to age-related fluctuations in the gut microbiome (Fig. 2a). Relative to the FF diet, SC1 and SC2 tended to select for taxa involved in degradation of dietary fiber, including Prevotellaceae, Lachnospiraceae, Muribaculaceae, and *Eubacterium* [62, 63] (Fig. 2b). Conversely, the FF-fed mice demonstrated an enrichment of known or potential mucin-degrading taxa, including Ruminococcaceae and *Akkermansia* [64, 65] (Fig. 2b). Fiber-deprived mice were characterized by an increase in mast cells, CD11c^+^ cells total ILCs, total T cells, CD62L^+^CD44^+^ T cells, LyC6^+^CD8^+^ T cells (CD44 +), and CD4^+^ T cells (Fig. 2c–d). Although total regulatory FoxP3^+^ T cells (Tregs) were similarly distributed between diets (Supplementary Table 4), SPF FF-fed mice presented with an increase in Tbet^+^ and RORgt^+^ Tregs, relative to SC1-fed mice (Fig. 2d).

### Custom diets on fiber-free background reveal effects of distinct fiber types

**Fig. 3:**
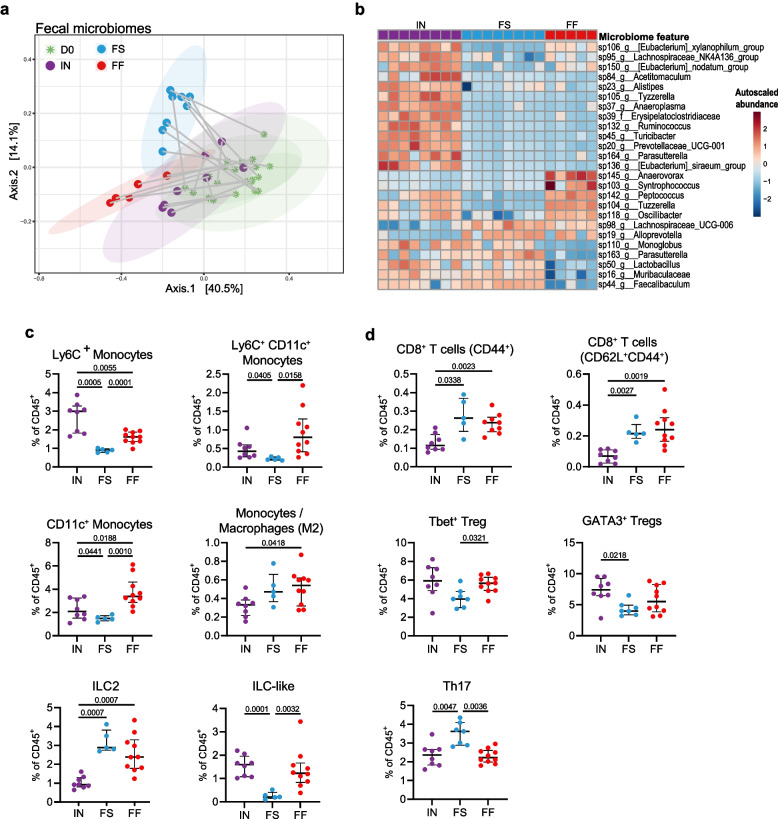
Fiber-supplemented diets differentially alter gut microbial and immune profiles in mice with complex microbiome. **a** Principal coordinates of analysis (PCoA) based on Bray–Curtis dissimilarity index from fecal microbiota of SPF mice at day 0 (D0, stars, light green) on standard chow and after feeding on an inulin-supplemented (IN), crude raw fiber-supplemented (FS), or fiber-free (FF) diet for 40 days (D40, dots, purple, blue, and red, respectively). Ellipses show 95% confidence interval (CI). **b** Heatmap of top 25 differentially abundant taxa in mice fed IN, FS, or FF diets according to a linear mixed-effect model with the FF group as reference. Microbiome feature abundances were normalized by total sum scaling and log-transformed, then auto-scaled for each taxa by mean-centering and dividing by standard deviation. Feature order is based on Ward clustering; sample order is fixed by group. For **a** and **b**, *n* = 5 (FF; one batch) or 8 (IN and FS; two batches of four mice) mice per group. Adaptive and innate immune clusters of the cLP, represented as % of total CD45^+^ cells, showing significantly different proportions among **c** innate or **d** adaptive immune populations (FF data same as in Fig. 1). CD4^+^ T cell were manually gated in FlowJo. No outliers were detected based on ROUT (*Q* = 1%). Statistical testing was performed using Brown–Forsythe and Welch ANOVA or Kruskal–Wallis test with *P* values adjusted using the Benjamini–Hochberg method. For **c**–**d**, *n* = 8 (IN: two batches of four mice each), 7–9 (FS: two batches of two to four mice, two samples that were excluded from cluster gating due to low cell count were included in the manual gating after confirming proportions not biased by low cell count), or 10 (FF: two batches of five mice) mice per group. Only populations with significant differences between the diets are shown; see Supplementary Table 3 for a complete list of populations analyzed

We observed distinctive effects of fiber deprivation on the host immune profile; however, the two standard chows and the custom fiber-free diet differ in a number of other macro- and micronutrient categories, including fat and protein content and sources (Supplementary Table 1). In order to isolate the effects of fiber alone, we commissioned the manufacture of two prebiotic-supplemented diets using the FF diet as a base (Supplementary Table 1). The first diet was supplemented with inulin (IN) by replacing 10% of the dextrose with the purified prebiotic fiber, inulin (ACROS Organics, Thermo Fisher Scientific), which contains polymers of fructose joined by β(2 → 1) glycosidic bonds that can be hydrolyzed by bacterial enzymes to yield fructo-oligosaccharides. In the second fiber-supplemented (FS) diet, 10% of the dextrose from the FF diet was replaced with a mix of equal parts of the following five concentrated raw fibers: apple, wheat, oat, pea, and psyllium [66] (J. Rettenmaier & Sohne, Germany). This diet gives the microbiota and host access to multiple prebiotic fibers, including pectin, beta-glucan, arabinogalactan, and arabinoxylan, and galacto-oligosaccharides. After 40 days of feeding on these diets, 16S rRNA gene sequencing of fecal samples revealed distinct clustering of the microbiome from mice fed FF, FS, or IN diets (Fig. 3a). Compared to FS-fed mice, the microbiome of IN-fed mice was strongly enriched in butyrate-producing bacteria, including three features from the genus *Eubacterium* (Fig. 3b).

We then assessed the broad immune phenotypes of both FS and IN-fed mice using CyTOF and compared the results with the FF-fed group from Fig. 2. Relative to the FF diet, IN and FS supplementation similarly demonstrated decreased proportions of CD11c^+^ monocytes (Fig. 3c), in addition to a number of shifts that were specific to the fiber type. Among IN-fed mice, this included an elevation of Ly6C^+^ monocytes, reduction of M2 monocytes/macrophages, and reduction of ILC2 populations among innate cell types (Fig. 3c), as well as a reduction of CD8 + T cells (CD44^+^ and CD62L^+^CD44^+^) among adaptive populations (Fig. 3d), compared to the FF group. The FS-fed mice demonstrated a reduction of Ly6C^+^ and Ly6C^+^CD11c^+^ monocytes along with a cluster characterized by ILC-like features (precise marker expression in Supplementary Fig. 1) among innate populations (Fig. 3c) and a reduction in Tbet^+^ Tregs and increase in Th17 cells among adaptive populations (Fig. 3d).

In peripheral organs, we also observed variations in immune cell profiles according to diet. In particular, splenic Th and Treg subsets appeared to vary according to the fiber type, with Th17 cells, Tbet^+^ Tregs, and RORgt + Tregs enriched in the IN group, whereas Th2 cells and GATA3^+^ Tregs were enriched in SC2 and FS groups (Supplementary Fig. 2c). Expression of either of the transcription factors T-bet or GATA-3 within the regulatory T cell pool have been described as being induced to control type 1 and type 2 inflammation, respectively [67, 68]. In the lung, SPF and GF mice fed an SC1 or FF diet were profiled, revealing elevated levels of total T cells and various subsets (i.e., CD44^−^CD62L^+^ , CD5^+^, and CD8^+^) among fiber-deprived SPF mice (Supplementary Fig. 3). Together, these data further support a strong effect of distinct plant polysaccharides and linkages, and thereby metabolic output, on both the microbiome composition and the host immune system.

### Short- and branched-chain fatty acids reflect diet-based clustering of microbiome features

**Fig. 4:**
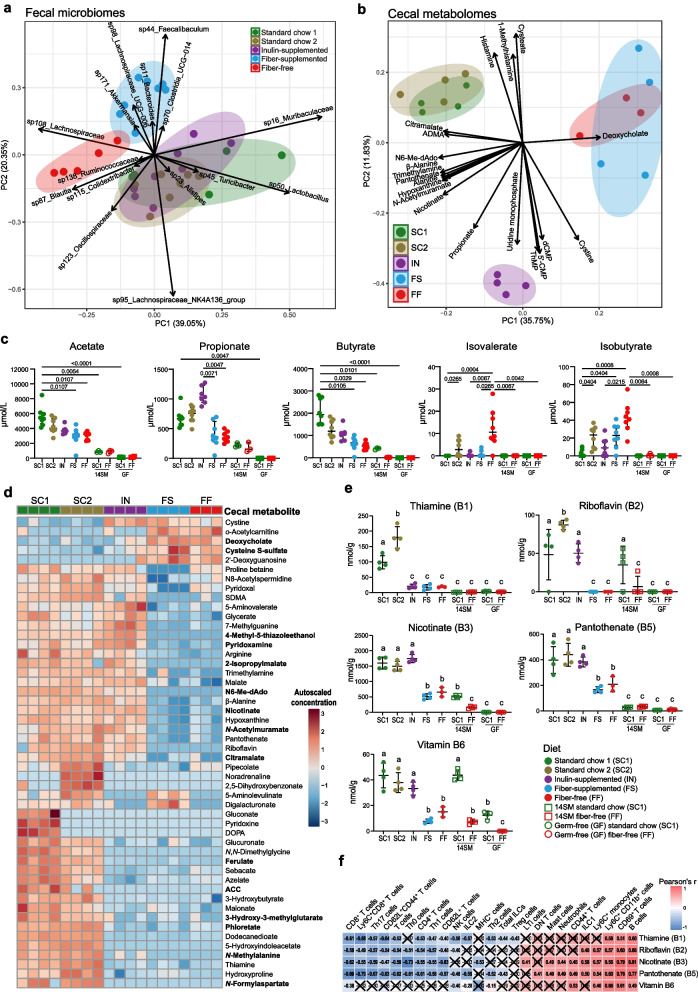
Inulin supplementation shifts microbiome and cecal metabolite profiles to resemble standard chow-fed mice. **a** PCA based on Bray–Curtis dissimilarity of the SPF fecal microbiome composition was generated for the five diet groups using samples from day 0 (all mice on SC1) and after 40 days of feeding on their respective diets (D40). Plot is scaled and centered with top 15 loadings are displayed. Ellipses represent 95% CI. *n* = 5–8 mice per group. **b** PCA of SPF cecal metabolomes for the five diet groups quantified by capillary electrophoresis time-of-flight mass spectrometry (CE-TOFMS), with loadings overlain for the top 20 metabolites contributing to PC1 and PC2. **c** SCFA and BCFA concentrations from cecal contents as quantified by GC–MS, adjusted by the initial sample weight of sample. One-way ANOVA with *P* values adjusted using the Benjamini–Hochberg method. Standard chow 1 (SC1) = green, standard chow 2 (SC2) = brown, inulin-supplemented (IN) = purple, fiber-supplemented (FS) = blue, fiber-free (FF) = red. **d** Heatmap of top 50 differentially abundant cecal metabolites in mice fed SC1, SC2, IN, FS, or FF diets according to a linear mixed-effect model with the FF group as reference. Metabolite concentrations were normalized to the cecal weight and log-transformed, then auto-scaled for each metabolite by mean-centering and dividing by standard deviation. Metabolite order is based on Ward clustering; sample order is fixed by group. Metabolites in bold were absent in germ-free samples. ACC, 1-aminocyclopropane-1-carboxylate; DOPA, 3,4-dihydroxyphenylalanine; N6-Me-dAdo, N6-methyl-2′-deoxyadenosine; SDMA, symmetric dimethylarginine; 3-HMG, 3-hydroxy-3-methylglutarate. **e** Concentrations of detected B vitamins (mean ± SD). Vitamin B6 includes pyridoxine, pyridoxal, pyridoxamine, and their phosphorylated derivatives. One-way ANOVA with adjusted *P* values calculated using the Benjamini–Hochberg method. Different letters denote statistically significant differences (adjusted *P* < 0.01). *n* = 4 mice per group (exception: one outlier removed from FF group based on PCA). **f** Correlation matrix between log-transformed cecal B vitamin concentrations and immune cell frequencies as a percent of CD45^+^ cells. Correlations that were not statistically significant (adjusted *P* ≥ 0.05) are indicated with an ex (×) over the Pearson correlation coefficient in each square. Data for correlations were derived from matched values (i.e., from the same mouse) for 14SM, GF, and SPF mice. Matched values were not available for SPF SC1, FS, and FF mice; therefore, the data was pooled for each of these groups

The microbial profiles of the SC1, SC2, and IN diets were largely overlapping and their clustering according to the Bray–Curtis dissimilarity metric was defined by increases in taxa such as Muribaculaceae, *Lactobacillus*, *Turicibacter*, *Alistipes*, Lachnospiraceae NK4A136 group, and Oscillospiraceae. Although we predicted that fiber deprivation would result in a reduction of bacterial diversity, the SC1 and FF diets demonstrated comparable alpha diversity according to multiple measures of richness (i.e., number of observed features, Faith’s phylogenetic diversity) and evenness (i.e., Shannon entropy, Pielou evenness) (Supplementary Table 6). Compared to the other standard chows (SC1 and SC2) or fiber-supplemented (IN and FS) diets after 40 days of feeding, FF-fed mice tended to exhibit impairment in SCFA production [62, 69, 70] (Fig. 4c), as expected in the absence of dietary fiber. Consistently, cecal acetate, butyrate, and propionate were decreased in FF group compared to SC1, as measured using GC–MS (Fig. 4c). By contrast, isobutyrate and isovalerate, branched-chain fatty acids (BCFAs) derived from the microbial metabolism of branched-chain amino acids, were increased in FF-fed mice (Fig. 4c). The observed changes in the microbiota and the related metabolites are in alignment with our previous findings in C57BL/6 mice fed the same FF diet [30], yet did not fully explain the observed differenced in the immune profiles. We therefore searched the cecal metabolome more broadly (detection of over 200 metabolites), in order to identify associations between the concentrations of specific metabolites and the proportions of distinct immune cell populations across the diets.

### Dietary fibers alter microbial B vitamin availability in the colon

In addition to SCFAs, gut bacteria produce a large spectrum of diet-derived metabolites with immuno-regulatory properties [9, 71, 72]. After observing distinct microbiota-mediated immune shifts due to changes in dietary fiber sources and/or structures, we aimed to determine additional metabolites potentially contributing to such immune changes using capillary electrophoresis time-of-flight mass spectrometry (CE-TOFMS)-based metabolome profiling from both cecal contents and serum of all five diet groups for SPF mice (SC1, SC2, FF, FS, and IN) and two dietary groups for GF and 14SM gnotobiotic mice (SC1 and FF) (Supplementary Tables 7–9). According to a pairwise adonis test [36], metabolome profiles of SC1 and SC2 significantly differed from FF and FS, which were distinct from each other as well as from IN (Fig. 4b). The IN metabolome was uniquely characterized by increases in metabolites involved in pyrimidine metabolism, e.g., uridine monophosphate and cytidine 5'-monophosphate (5′-CMP) (Fig. 4b).

We then compared the cecal metabolomes with their respective serum metabolomes and observed that the total number of metabolites detected in both the cecum and the serum was relatively consistent across all diets, although the ratio of unique metabolites (cecum vs serum) was lowest for fiber-deprived mice (Supplementary Fig. 4a). Nonetheless, PCA analysis of the serum metabolomes did not distinguish statistically significant clustering between diets using a pairwise adonis test (Supplementary Fig. 4b). We then considered the top (lowest *P* value) 50 cecal metabolites as assessed by ANOVA that show unique diet-specific clustering (Fig. 4d, bold metabolites were not detected in GF controls). To identify microbiota-dependent metabolites within the cecal contents, we additionally analyzed the metabolome of GF and 14SM gnotobiotic mice fed either SC1 or FF (Supplementary Fig. 5). GF and gnotobiotic SC1- and FF-fed mice displayed distinct metabolomes, with a striking reduction in unique metabolites among germ-free mice compared to 14SM or SPF mice (Supplementary Fig. 5a), including 98 metabolites that were only detected in colonized (i.e., inclusive union of SPF and 14SM sets) SC1-fed mice and 66 in FF-fed mice (Supplementary Fig. 5c).

In addition to SCFA production, the gut microbiota is also known to regulate bile acid synthesis and metabolism [73–75]. Here, we also found significant differences in serum and cecal bile acid concentrations that were both diet- and microbiome-dependent (Supplementary Fig. 4a–c). Primary bile acids taurocholic acid (TCA) and glycocholic acid (GCA) are produced in the liver and secreted into the small intestine to emulsify fats [73]. The collective elevation of serum and cecal bile acids in IN, FS, and FF diets (Supplementary Fig. 4d–e) may be reflective of their uniformly higher fat content (15.3% by weight) compared to the SC1 and SC2 diets (3.1% and 5.1%, respectively) (Supplementary Table 1). Furthermore, an animal-based protein diet is rich in taurine and tends to yield higher amounts TCA, which is supported in Supplementary Fig. 4e, with the FF diet yielding more TCA in GF and 14SM mice. Approximately 5–10% of these primary bile acids pass into the lower GI tract, where microbes can metabolize them into secondary bile acids such as deoxycholic acid (DCA), by first removing the glycine or taurine moiety to form cholic acid (CA) [73]. Accordingly, neither CA nor DCA were detectable in the cecal contents of GF mice (Supplementary Fig. 4e), consistent with previous observations of microbiome-dependent bile acid metabolism [74, 76]. Secondary bile acids have important regulatory effects on mucosal immunity; however, microbial production of DCA yields hydrogen sulfide as a byproduct, which can promote inflammation [77, 78]. DCA, which is detected by CETOF-MS in the conjugate base form and therefore reported as deoxycholate, was the main feature driving separation among the cecal metabolome between FF/FS and SC1/SC2/IN (Fig. 4b).

Overall, both the fecal microbiomes (Fig. 4a) and the cecal metabololomes (Fig. 4b) showed similarities between the IN-fed group with SC1- and SC2-fed groups. Among metabolites that were commonly increased in these three groups, we noted an increased concentration of B vitamins in SC1, SC2, and IN groups, which were either decreased or absent in FF- and FS-fed mice (Fig. 4e). Specifically, concentrations of riboflavin (vitamin B2), nicotinate (vitamin B3), pantothenate (vitamin B5), and vitamin B6 were higher in SC1, SC2, and IN groups and were almost absent from FS and FF groups. Meanwhile, thiamine (vitamin B1) was increased in SC1- and SC2-fed mice compared to all other dietary groups (Fig. 4e). We were unable to detect riboflavin nor nicotinate in either dietary groups of GF mice (Fig. 4e), in line with other published metabolome analyses reporting relative fold changes in conventionally colonized versus GF mice [72]. With the exception of vitamin B6 in 14SM SC1-fed mice, the concentrations of B vitamins were low compared to SPF SC1-fed mice (Fig. 4e). Importantly, serum B vitamin concentrations did not differ significantly between the diet groups, underscoring the importance of gut microbial B vitamin production in lower GI tract to shape local immunity (Supplementary Table 9). These results support a model that the metabolism of dietary fibers by the gut microbiota boosts local B vitamin production, considering B vitamins were largely absent in both GF dietary groups and the concentration of the B vitamins was dependent on the presence of both the appropriate fiber sources and the complexity of the gut microbiota (Fig. 4e, Supplementary Table 7). Concentrations of B vitamins showed negative correlations with pro-inflammatory innate and adaptive immune cell frequencies, especially for mast cells, NK cells, and Th17 cells (Fig. 4f).

**Fig. 5:**
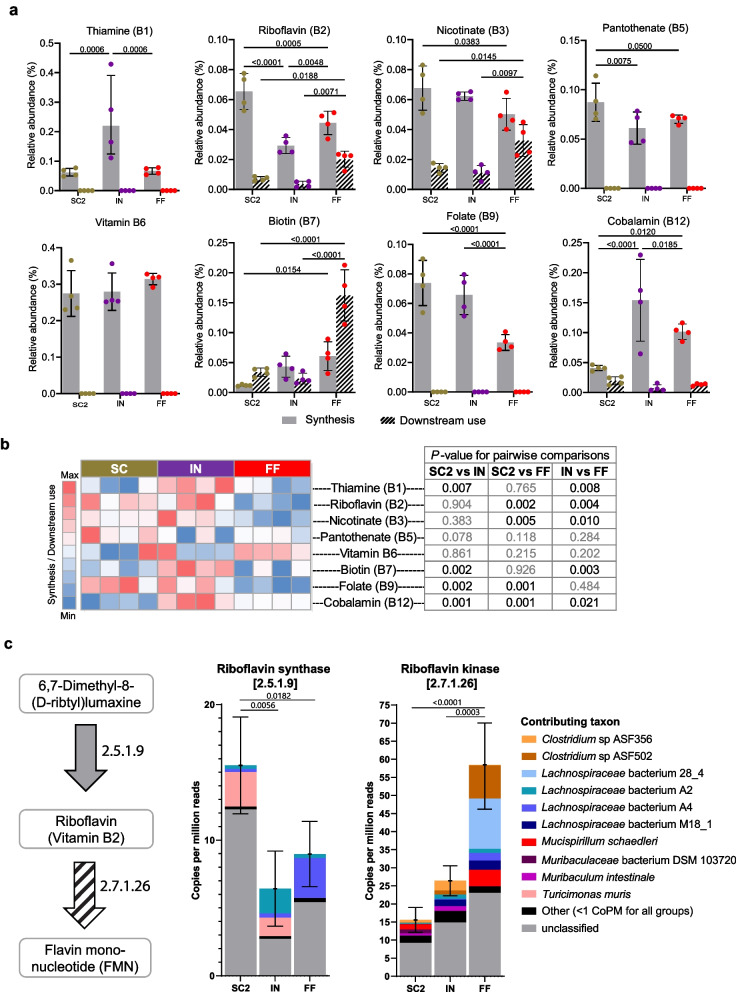
B vitamin synthesis and utilization dynamics are disrupted in fiber-deprived conditions. **a** Relative abundance (%) of cecal transcripts mapping to non-host genes involved in B vitamin synthesis or downstream utilization. One-way ANOVA with adjusted *P* values calculated using the Benjamini–Hochberg method. **b** Log2-adjusted ratio of all upstream synthesis over downstream utilization transcripts (S/D) for B vitamins in cecal metatranscriptomes for mice fed a standard chow (SC2), an inulin supplemented diet (IN), or a fiber-free (FF) diet. Pseudocounts (plus one to all values) were used when downstream values were 0 (e.g., thiamine, pantothenate, vitamin B6, and folate) to permit the calculation of log2-adjusted ratios. Color intensity is relative to min and max within each row, with the midpoint at the median value. Unpaired *t* test, non-significant (*p* > 0.05) values in gray. **c** Bacterial contributors for enzymes immediately up- or downstream of riboflavin (vitamin B2), based on the KEGG riboflavin metabolism reference pathway [79]. *n* = 4 mice per group, mean ± SD

The novel link between levels of B vitamin availability in the lower GI tract and dietary fiber consumption prompted us to investigate whether gut microbes involved in fiber metabolism directly produce B vitamins or whether the overall fiber metabolism by the gut microbiota alters the status quo such that more B vitamins are available for both the microbiota and the host. In order to determine the role of the microbiota in B vitamin availability, we assessed B vitamin synthesis and utilization dynamics in the cecal metatranscriptomes of IN-, SC2-, and FF-fed SPF mice. Based on the metabolome data, we expected to find a reduction in transcripts corresponding to genes involved in B vitamin synthesis in the FF-fed mice; however, we noted that the production was variably impacted across diets, only consistently decreased in FF relative to IN and SC2 for folate (vitamin B9) (Fig. 5a). Intriguingly, transcripts encoding enzymes involved in downstream utilization of these B vitamins were more highly expressed in FF-fed mice for riboflavin (vitamin B2), nicotinate (vitamin B3), and biotin (vitamin B7), suggesting differential metabolic needs of the gut microbiota under fiber-deprived conditions detract from the pool of B vitamins available for host uptake. This finding is best summarized by considering the ratio of upstream synthesis relative to downstream utilization transcripts for B vitamins in each group (Fig. 5b). The log2-transformed ratio of transcripts involved in synthesis versus downstream use was significantly higher for thiamine, riboflavin, nicotinate, biotin, and cobalamin in the IN to FF comparison and for riboflavin, nicotinate, folate, and cobalamin in the SC2 to FF comparison (Fig. 5b).

**Fig. 6:**
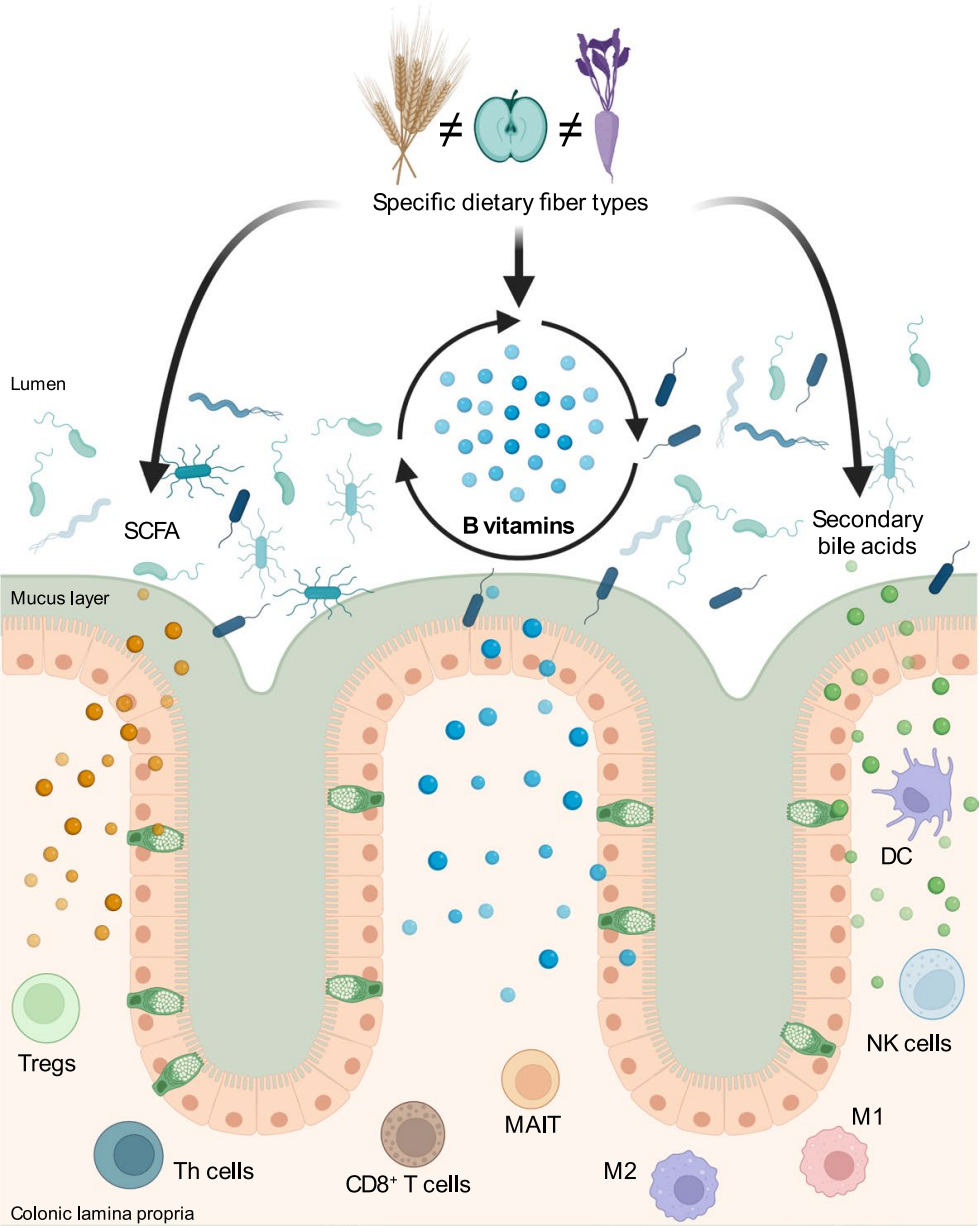
Summary of diet–microbiome–immune interactions in the colon. Dietary fibers of diverse plant origins are metabolized by the gut microbiome to produce short-chain fatty acids, which support Tregs. B vitamins are both synthesized and utilized by the gut microbiome and impact diverse immune cell subsets as reviewed by Gholami et al. [82]. Secondary bile acids are also produced by the microbiome and show variation by diet, with distinct immune impacts as reviewed by Fiorucci et al. [75]. Figure created with BioRender.com

Furthermore, the bacteria responsible for carrying out the synthesis or degradation were distinct between groups fed fiber-containing (IN and SC2) and fiber-deprived (FF) diets (Fig. 5c, Supplementary Fig. 6, 7), reflecting the taxonomic diversity observed in 16S rRNA gene sequencing analysis (Fig. 4a, b) and the predicted composition according to the metatranscriptome (Supplementary Fig. 6a). While not all transcripts corresponding to enzymes immediately up- or downstream of each B vitamin could be uniquely attributed to a specific bacterium (i.e., “unclassified” contributing taxon), we note that *Mucispirillum schaedleri—*a mucus-associated bacterium—and *Clostridium* sp. ASF502—a close relative of the mucin-degrading bacterium *Ruminococcus gnavus*—were key contributors to the downstream utilization of these vitamins in FF-fed mice (Fig. 6c, Supplementary Fig. 6–7). Both strains are members of the altered Schaedler flora (ASF) community [80], a well-studied gnotobiotic model; nonetheless, based on our findings, we would speculate that this normally stable community is altered by fiber deprivation. Similarly, several bacteria belonging to the Lachnospiraceae family, including strains A4 and 28–4, were contributors of transcripts of enzymes downstream of riboflavin and nicotinate. These bacteria are associated with TGF-β production and inhibition of Th2 in the cLP in vivo [81]; however, cultivable isolates have not been identified and thus their specific functions have not been validated.

## Discussion

In this study, we assessed the effect of dietary fiber deprivation and supplementation across the microbiome–metabolome–immune axes. Using 16S sequencing, CE-TOFMS metabolomics, CyTOF immunophenotyping, and metatranscriptomic analyses, we provide a comprehensive view of pathways directly or indirectly regulated by the metabolic products of dietary fiber fermentation by the gut microbiota. We show that, while dietary fiber deprivation drives an inflammatory gut environment, not all sources of fiber are equal in their capacity to maintain a homeostatic state at the microbial, metabolic, and immune levels. Importantly, we identify a potential role of gut microbial production of B vitamins in maintaining immune homeostasis, which is lost during fiber deprivation. Our results suggest that, in addition to its previously described impact through microbiota-mediated mucus erosion [22, 30, 83, 84], a fiber-deprived diet disrupts the gut microbiome’s metabolic output, contributing to broad activation of local immune populations (Fig. 6).

Metabolomics analysis revealed a microbiome-mediated loss of several barrier-protective and immunoregulatory metabolites such as SCFAs and B vitamins in the cecum of FF-fed mice (Figs. 4, 5). Although it is well-understood that SCFAs are the end products of fiber fermentation, our data establish a novel association between B vitamins and dietary fiber. B vitamins are involved in many host biochemical and metabolic pathways and also display important immunomodulatory properties [14, 71, 85]. Thiamine (vitamin B1) is a critical cofactor in the tricarboxylic acid (TCA) cycle, which is preferentially used by naïve and regulatory immune cells for energy [10]. While a decrease in the availability of this vitamin would not be expected to impact differentiation, it may impair the maintenance of these non-activated populations, as demonstrated for naïve B cells among mice fed a B1-deficient diet [86]. Riboflavin (vitamin B2) also has anti-inflammatory properties systemically [87], primarily through reducing oxygen in the gut, allowing for growth of host-beneficial fiber-degrading bacteria [88]. Intriguingly, Krause et al. identified a positive relationship between the microbial biosynthesis of riboflavin and activation of mucosal-associated invariant T (MAIT) cells, which, in line with our findings, was impaired under acid stress conditions due to high microbial utilization of riboflavin [89]. Nicotinate (vitamin B3), like the SCFA butyrate, signals through GPCR109a [90], suppressing the production of proinflammatory cytokines and inducing Treg cells in the cLP [91]. Serum nicotinamide, the absorbed form of vitamin B3, was highest among FF-fed mice (Supplementary Table 9), which corroborates the observation by Blacher et al. that *Akkermansia muciniphila* is associated with increased nicotinamide [92], as SPF and 14SM FF-fed mice exhibited elevated abundances of this mucin-degrading bacterium. Paul et al. demonstrated that pantothenate (vitamin B5), a precursor of coenzyme A, promotes CD8^+^ Tc22 polarization to enhance anti-tumoral immunity [93]. Another study found that supplementation with pantothenate during *Mycobacterium tuberculosis* infection had a strong inflammatory effect on macrophages leading to pathogen clearance [94]. Vitamin B6, which includes pyridoxine, pyridoxal, pyridoxamine, and their phosphorylated derivatives, exhibits anti-inflammatory properties when supplemented in multiple immune disorders, including arthritis [95] and colon cancer [96]. Vitamin B6 also protected mice from endotoxic shock by decreasing IL-1β production and further inhibiting NLRP3 inflammasome activation [97]. In our study, vitamin B6 was elevated in mice fed the SC1, SC2, and IN diets (Fig. 5). Although the increased activation state in FF mice was not inherently negative in our study, i.e., mice did not show overt signs of intestinal inflammation at the end of the protocol, these metabolic shifts could alter the host response upon challenge with a pathogen or allergen and/or make the host more prone to developing autoimmune diseases by exerting a chronic negative strain on the immune system. Furthermore, considering GF SC1- and FF-fed mice did not exhibit significant differences in immune cell frequencies (Fig. 1), B vitamin production by the gut microbiota may be an important contributor to the immunological changes that we identified in colonized mice.

Previous studies have identified dietary fiber as a key driver in microbiome and immune homeostasis [8, 98]. Interestingly, supplementation of the FF diet with inulin (IN), but not crude fibers (FS), was able to boost local immune regulation by increasing Tregs and decreasing effector cell populations (Fig. 3). Although whole fibers have been shown to have human health benefits [99], with psyllium supplementation alone reducing colitis severity in SPF mice [100], we were unable to identify significant immune or metabolic benefits in our FS diet, which contains highly concentrated forms of such fibers. There are a number of possible explanations for why this diet did not have a pronounced effect. Although the intestinal layout between mice and man consists of similar organs, the murine digestive tract is considerably shorter than that of humans. Transit time has been extensively studied in vitro, revealing significant alterations to both microbiome composition, metabolic capacity, and fermentation efficiency [101, 102]. The intestinal transit time in mice can be up to 10 times faster than humans and the substantially larger amount of time available for bacterial fermentation in humans potentially allows for fermentation of specific fiber types, such as those in the FS diet [103, 104]. The microbiome of an SPF mouse also differs from that of a human, most notably due to our differing diets. In this case, it is feasible to consider that in a microbiome lacking the ability to degrade specific carbohydrate linkages or without adequate fermentation time, the concentrated raw fibers originally designed for human supplementation would be inaccessible to the mouse host. However, we did observe differences between FS- and FF-fed mice in the microbial profiles and for specific immune data (Fig. 3, Supplementary Figs 2 and 3), indicating that the FS diet did have some host effect, albeit minimal compared to IN-fed mice.

The two standard chows serve as a reference for a healthy host immunophenotype; however, there are other dietary components beyond fiber that differ between these chows. It is generally important to recognize that standard chows (SC1 and SC2 in this study) can have different formulations that contribute to diverse colonic immunophenotypes. One strength of our approach lies in the use of custom fiber-enriched diets on the FF background diet to isolate the effects of fiber alone. The custom IN, FS, and FF diets were all produced in the same manufacturing facility, with the same foundational ingredients, including vitamin mix, thus excluding bias from variations in dietary vitamin concentration (Supplementary Table 1). Moreover, dietary B vitamins are primarily absorbed in the proximal part of the small intestine with excess—aside from cobalamin (vitamin B12)—excreted in the urine [105]. In germ-free mice fed an SC1 or FF diet, B vitamins in the cecal metabolome were extremely low or undetectable, compared to SPF mice (Fig. 4e), indicating that the B vitamins measured in this study were largely produced by the gut bacteria and were not diet-derived. This observation is in line with the recently published findings of increased fold change of thiamine, riboflavin, nicotinate, and vitamin B6 in conventionally colonized versus germ-free mice [72]. Together, these points provide strong evidence that the elevated levels of B vitamins in the lower half of the GI tract in colonized mice are due to the gut microbiota and that the reduction in the pool of B vitamins under fiber-deprived conditions is due to altered microbial metabolism.

A key finding of this study was the role of standard lab chows and inulin supplementation on microbial B vitamin availability, in contrast to the crude fiber-supplemented and fiber-free diets. In a high-fat diet mouse model, supplementation with fructo-oligosaccharides—inulin-type fructans—and vitamin B9 (biotin) improved the potential of bacterial production of B vitamins, with positive host health outcomes [106]. Although it is known that microbes can biosynthesize B vitamins to support immune homeostasis [82], our study contributes a new lens to this dynamic by providing evidence that dietary fiber can shape the gut environment not by supporting the growth of B vitamin synthesizers, but rather by limiting the proliferation or altering the metabolic activities of bacteria that use B vitamins for downstream processes to detract from the host-available pool for certain B vitamins, including riboflavin, nicotinate, and biotin. In this model, inulin supplementation sufficiently recovers the phenotype of an SC1- or SC2-fed mouse by promoting a microbiome that disfavors use of B vitamins for downstream processes, which further restores an immunoregulatory state, both of which are lost during fiber deprivation. Thus, while it is useful to catalogue bacteria that can produce B vitamins within the gut microbiome [10], our findings suggest that these bacteria are inappropriate targets to counteract B vitamin deficiencies and offer a possible explanation of previous null findings when addressing potentially disease-related vitamin deficiencies via supplementation [107]. Rather, we suggest developing approaches targeting the members of the community that consume these B vitamins, especially through dietary modulation to alter their abundance or metabolism.

While we did not employ a disease model in the present study, a possible extension of these findings would be to examine the potential to alter the immune response in disease by augmenting the microbial metabolism of B vitamins. Supplementation with specific fibers, such as psyllium, was shown to improve survival in a DSS-induced colitis mouse model [100], while inulin, when supplemented to a high-fat diet was shown to exacerbate disease [108]. The exclusion of dietary fiber is also detrimental in the same disease model [109]; therefore, it remains necessary to consider which specific types of fiber can be employed in various disease contexts. To this end, a basal understanding of the immune shifts induced by specific fibers in a healthy, homeostatic state is a necessary precondition to determine their appropriate implementation to counter disease, either in therapies or as preventive measures [110].

## Conclusions

These data emphasize the multimodal role of dietary fiber to harness microbial synthesis of anti-inflammatory and immune-regulating metabolites and vitamins in the colon, as summarized in Fig. 6. Importantly, our data support previous findings that SCFAs are key immune regulators, but the microbial metabolome has many more actors that need to be further understood in the context of health and disease. Using custom fiber-supplemented diets in a mouse model, we demonstrated that not all fibers have the same capacity to restore host–immune homeostasis, indicating that specific dietary fibers supplements should be selected based on an individual microbiome’s capacity to degrade specific linkages. While we highlight dynamics for B vitamins in this work, it is also clear that the synthesis of other microbial metabolites, such as secondary bile acids and the expected SCFAs, are also altered in the face of fiber deprivation, collectively influencing local immunity. Assessing metabolic products from the fermentation of specific fiber types by specific bacterial species will be a useful intermediate step in translating this work to humans, along with mechanistic work in cell lines or mice to improve understanding of the associated host effects. Follow-up studies in humans are essential to capitalize on these early associations and aid in the development of personalized dietary interventions, which can be tailored to an individual microbiome. These translational efforts should focus on the development of prebiotic fibers that boost the local availability of immunomodulatory metabolites in the lower GI tract, including, but not limited to, B vitamins, by considering microbial activity and metabolic output under various environmental conditions, rather than focusing on taxonomic composition or functional capacity. Ultimately, this information could be leveraged to promote a gut environment and microbiome with a higher net output of immunomodulatory metabolites to support host health.

## Supplementary Information

Supplementary Material 1: Supplementary Figure 1. Annotated cluster heatmap of colonic lamina propria (cLP) analysis. CD45^+^ single live cells were imported into R for FlowSOM analysis. Based on the expression of 26 markers columns), 100 clusters were generated and manually merged based on marker expression (columns) to form 30 biologically relevant cell populations (rows). Percentages in parentheses next to the population name represent the percentage of that population among CD45^+^ cells. Marker expression is normalized between 0 and 1. Supplementary Figure 2. CyTOF analysis reveals systemic immune changes from a fiber-free diet. a) UMAP visualization of CD45^+^ immune cells isolated from the spleens of SPF (top) and GF (bottom) mice, alongside the markers used to elucidate main cell subsets (all groups pooled). c) Select marker expression facetted by colonization state and diet. Samples within each group were downsampled and then concatenated such that 60,000 events were analyzed per group (420,000 total). c) Splenic immune cell populations significantly different between specific-pathogen-free (SPF) mice fed a standard chow 1 (SC1), standard chow 2 (SC2), inulin-supplemented (IN), fiber-supplemented (FS) or fiber-free (FF) diet, alongside germ-free (GF) mice fed an SC1 or FF diet, represented as % of total CD45^+^ cells. Statistical testing was performed using Brown-Forsythe and Welch ANOVA or Kruskal-Wallis test with *P* values adjusted using the Benjamini–Hochberg method. *n*=4 (SPF SC1), 8 (SC2, IN, FS; 2 batches of 3–4 mice), 5 (SPF FF), or 6–7 (GF SC1, GF FF; 2 batches of 3–4 mice) mice per group. Only populations with significant differences between the diets are shown; see Supplementary Table 4 for complete list of populations analyzed. For SC2 and FS, one sample per diet was not analyzed by CyTOF due to low viable cell count; for IN, one sample (In4) was excluded from subsequent analyses due to low event count. Supplementary Figure 3. CyTOF analysis reveals pulmonary immune changes from a fiber-free diet. a) UMAP visualization of CD45^+^ immune cells isolated from the lungs of SPF (top) and GF (bottom) mice, alongside the markers used to elucidate main cell subsets (all groups pooled). c) Select marker expression facetted by colonization state and diet. Samples within each group were downsampled and then concatenated such that 100,000 events were analyzed per group (400,000 total). c) Pulmonary immune cell populations significantly different between specific-pathogen-free (SPF) mice fed a standard chow 1 (SC1) or fiber-free (FF) diet, alongside germ-free (GF) mice fed an SC1 or FF diet, represented as % of total CD45^+^ cells. Statistical testing was performed using Brown-Forsythe and Welch ANOVA or Kruskal-Wallis test with *P* values adjusted using the Benjamini–Hochberg method. *n*=5 (SPF SC1, SPF FF) or 3 (GF SC1, GF FF) mice per group. Only populations with significant differences between the diets are shown; see Supplementary Table 5 for complete list of populations analyzed. Supplementary Figure 4. Comparison of serum and cecal metabolomes across diets. a) Venn diagrams and UpSet plot representing intersecting sets of metabolites present in serum and cecal contents of specific-pathogen-free (SPF) mice fed all diets: standard chow 1 (SC1), standard chow 2 (SC2), inulin-supplemented (IN), fiber-supplemented (FS) or fiber-free (FF). b) PCA of serum metabolomes with loadings overlain for the top 20 metabolites contributing to PC1 and PC2. Ellipses represent 95% CI. c) Heatmap of the 38 differentially abundant serum metabolites in SPF mice fed each diet according to a linear mixed-effect model with the GF FF group as reference. Concentrations were normalized by volume, log-transformed, then scaled for each metabolite by mean-centering and dividing by the standard deviation. Metabolite order is based on Ward clustering; sample order is fixed by group. d) Serum concentrations of primary bile acids taurocholate and free bile acid cholate. e) Cecum concentrations of primary bile acids taurocholate and glycocholate, free bile acid cholate, and secondary bile acid deoxycholate. One-way ANOVA with adjusted *P* values calculated using the Benjamini–Hochberg method. *n*=4 mice per group (one outlier removed from FF group based on PCA), mean ± SD. Different letters denote statistically significant differences (adjusted *P*<0.01). Standard chow 1 (SC1)=green, standard chow 2 (SC2)=brown, inulin-supplemented (IN)=purple, fiber-supplemented (FS)=blue, fiber-free (FF)=red. Supplementary Figure 5. Cecal metabolome of germ-free mice is less perturbed by diet. a) Venn diagrams and UpSet plot representing intersecting sets of metabolites present in cecal contents of specific-pathogen-free (SPF), gnotobiotic (14SM), and germ-free (GF) mice fed a standard chow 1 (SC1) or fiber-free (FF) diet. b) PCA of cecal metabolomes of GF and 14SM mice fed an SC1 or FF diet with loadings overlain for the top 20 metabolites contributing to PC1 and PC2. Ellipses represent 95% CI. c) Heatmap of top 50 differentially abundant cecal metabolites in GF and 14SM mice fed an SC1 or FF diet according to a linear mixed-effect model with the GF FF group as reference. Metabolite concentrations were normalized to the cecal weight, log-transformed, then scaled for each metabolite by mean-centering and dividing by the standard deviation. Metabolite order is based on Ward clustering; sample order is fixed by group. *n*=4 mice per group (one outlier removed from SPF FF group based on PCA). Standard chow 1 (SC1)=green, fiber-free (FF)=red, 14SM=open squares, GF=open circles. Supplementary Figure 6. Bacterial contributors to vitamin B1, B2, and B5 metabolism in the cecum. a) Predicted community composition based on metatranscriptome using MetaPhlan 3 within HUMAnN 3. b) Stratified abundance of enzymes immediately upstream of thiamine phosphate and thiamine pyrophosphate (vitamin B1 precursors). TMPase is produced by the host, therefore all bacterial transcripts shown here correspond to enzymes that contribute to thiamine availability. c) Stratified abundance of enzymes involved in nicotinate (vitamin B3) synthesis. d) Stratified abundance of enzymes immediately up and downstream of pantothenate (vitamin B5). *n*=4 mice per group, error bars represent SD. Supplementary Figure 7. Bacterial contributors to vitamin B6, B7, B9 and B12 metabolism in the cecum. a) Stratified abundance of enzymes immediately upstream of pyridoxine, pyridoxamine, and pyridoxal and their phosphorylated derivatives (vitamin B6). b) Stratified abundance of enzymes immediately up and downstream of biotin (vitamin B7). c) Stratified abundance of enzymes immediately upstream of folate (vitamin B9). d) Stratified abundance of enzymes immediately upstream of cobalamin (vitamin B12). *n*=4 mice per group, error bars represent SD.

Supplementary Material 2: Supplementary Table 1. Composition of rodent diets. Composition of all diets used in this study: standard chow 1 (SC1), standard chow 2 (SC2), inulin-supplemented (IN), fiber-supplemented (FS) or fiber-free (FF). Supplementary Table 2. CyTOF antibody panel. Antibody panel used to stain cells isolated from colonic lamina propria, lung, and spleen samples for CyTOF analysis. Supplementary Table 3: Colonic lamina propria CD45+ cell abundances by CyTOF. Colonic lamina propria immune cell populations acquired by CyTOF, expressed as a percentage of all CD45+ cells. Cell populations were identified by clustering in FlowSOM and by manual gating in FlowJo for CD4+ populations. N indicates number of samples included in the analyses; batch number indicates independent experiments for a given diet. Supplementary Table 4: Spleen CD45+ cell abundances by CyTOF. Splenic immune cell populations acquired by CyTOF, expressed as a percentage of all CD45+ cells. Cell populations were identified by manual gating in FlowJo. N indicates number of samples included in the analyses; batch number indicates independent experiments for a given diet. Supplementary Table 5: Lung CD45+ cell abundances by CyTOF. Lung immune cell populations acquired by CyTOF, expressed as a percentage of all CD45^+^ cells. Cell populations were identified by manual gating in FlowJo. N indicates number of samples included in the analyses; batch number indicates independent experiments for a given diet. Supplementary Table 6. Microbiome composition by 16S rRNA gene sequencing. Composition of fecal microbiota by 16S rRNA gene sequencing of specific-pathogen-free (SPF) mice before (day 0) and after 40 days of feeding (day 40) for all diets: standard chow 1 (SC1), standard chow 2 (SC2), inulin-supplemented (IN), fiber-supplemented (FS) or fiber-free (FF). Supplementary Table 7. Cecum metabolome of germ-free (GF) and gnotobiotic (14SM) mice. Concentrations of metabolites detected by CE-TOFMS in cecal contents of gnotobiotic (14SM), and germ-free (GF) mice fed a standard chow 1 (SC1) or fiber-free (FF) diet. Supplementary Table 8. Cecum metabolome of specific-pathogen-free (SPF) mice. Concentrations of metabolites detected by CE-TOFMS in cecal contents of specific-pathogen-free (SPF) mice fed all diets: standard chow 1 (SC1), standard chow 2 (SC2), inulin-supplemented (IN), fiber-supplemented (FS) or fiber-free (FF). Supplementary Table 9. Serum metabolome of specific-pathogen-free (SPF) mice. Concentrations of metabolites detected by CE-TOFMS in serum of specific-pathogen-free (SPF) mice fed all diets: standard chow 1 (SC1), standard chow 2 (SC2), inulin-supplemented (IN), fiber-supplemented (FS) or fiber-free (FF). Supplementary Table 10. B vitamin metatranscriptome. Relative abundance of transcripts mapping to bacterial genes involved in synthesis (S) or downstream utilization (D) of enzymes involved in B vitamin metabolism in cecal contents of specific-pathogen-free (SPF) mice fed an inulin-supplemented (IN), standard chow 2 (SC2), or fiber-free (FF) diet. Supplementary Table 11. Metatranscriptome mapped by Enzyme Classification (EC) identifiers. Transcripts mapping to bacterial genes with the specified Enzyme Classification (EC) identifier, expressed as counts per million reads, in cecal contents of specific-pathogen-free (SPF) mice fed an inulin-supplemented (IN), standard chow 2 (SC2), or fiber-free (FF) diet.

## Data Availability

The raw fastq files for this study from 16S rRNA gene sequencing and RNA sequencing have been deposited in the European Nucleotide Archive (ENA) at EMBL-EBI under accession number PRJEB51707 (https://www.ebi.ac.uk/ena/browser/view/PRJEB51707). The mass cytometry datasets for colonic lamina propria, lung, and spleen have been uploaded to the Zenodo data repository (10.5281/zenodo.13253855). Raw spectral data from CE-TOFMS are available under Project ID PR001381 on Metabolomics Workbench [111] (https://www.metabolomicsworkbench.org), which is the NIH Common Fund’s National Metabolomics Data Repository (NMDR), supported by NIH grant U2C-DK119886. The data can be accessed directly via the Project DOI: 10.21228/M86T35.

## Abbreviations

14SM: 14-Member synthetic human gut microbiota
BCFA: Branched-chain fatty acid
CA: Cholic acid
CETOF-MS: Capillary electrophoresis time-of-flight mass spectrometry
cLP: Colonic lamina propria
CyTOF: Mass cytometry by time-of-flight
DCA: Deoxycholic acid
FF: Fiber-free (diet)
SC1: Standard chow 1 (diet)
SC2: Standard chow 2 (diet)
FS: Fiber-supplemented (diet)
GCA: Glycocholic acid
GC-MS: Gas chromatography mass spectrometry
GF: Germ-free
GI: Gastrointestinal
IN: Inulin-supplemented (diet)
MAIT: Mucosal-associated invariant T (cells)
PCoA: Principle coordinates of analysis
rRNA: Ribosomal RNA
SCFA: Short-chain fatty acid
SPF: Specific-pathogen-free
TCA: Taurocholic acid
Treg: Regulatory T cells
UMAP: Uniform Manifold Approximation and Projection
