# Supplementary figures and images for: Dietary fibers boost gut microbiota-produced B vitamin pool and alter host immune landscape
Source: Microbiome. 2024 Sep 23;12:179. doi: 10.1186/s40168-024-01898-7 (PMC11418204; doi:10.1186/s40168-024-01898-7)

Fig S1

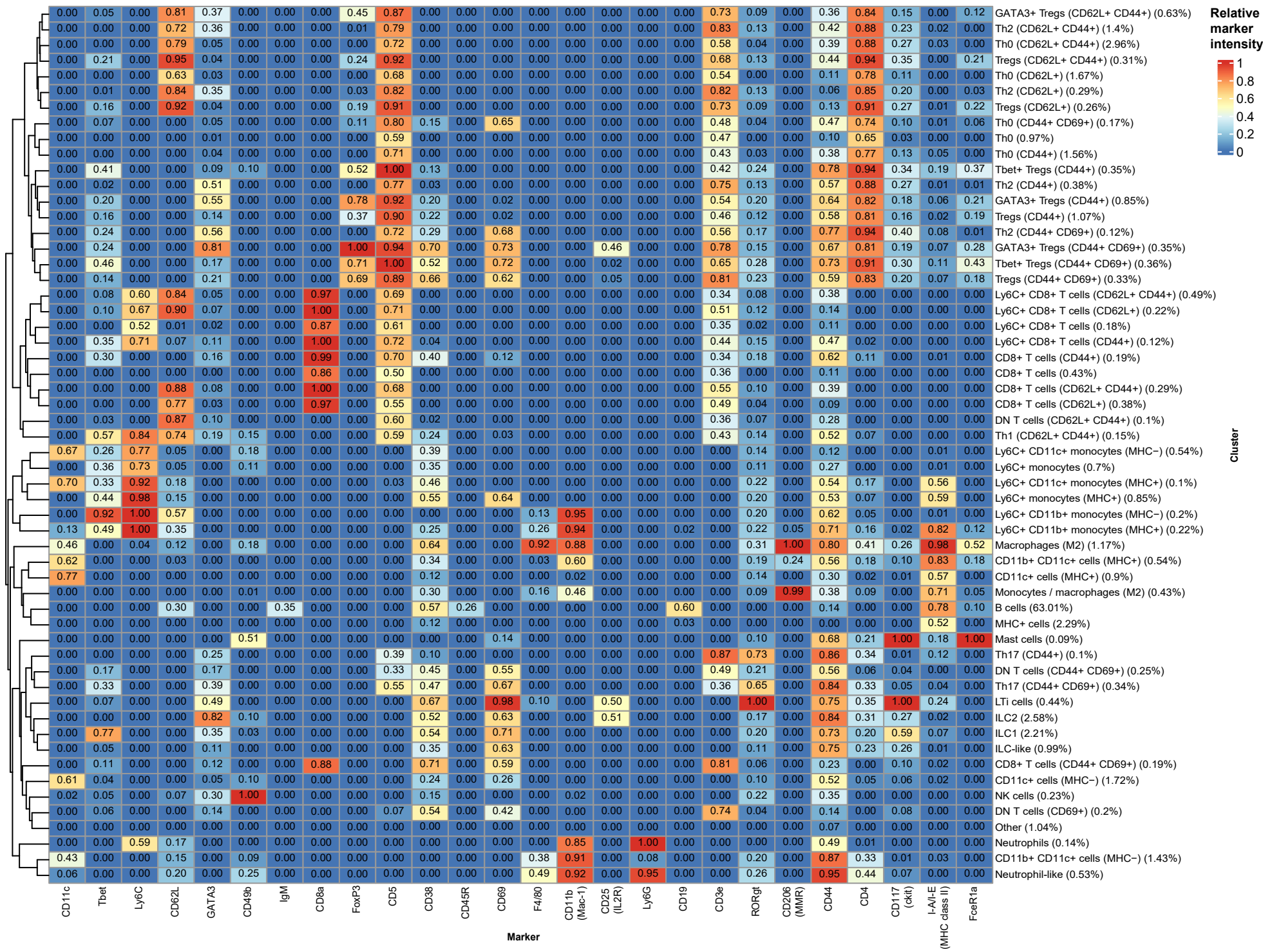

FigS2

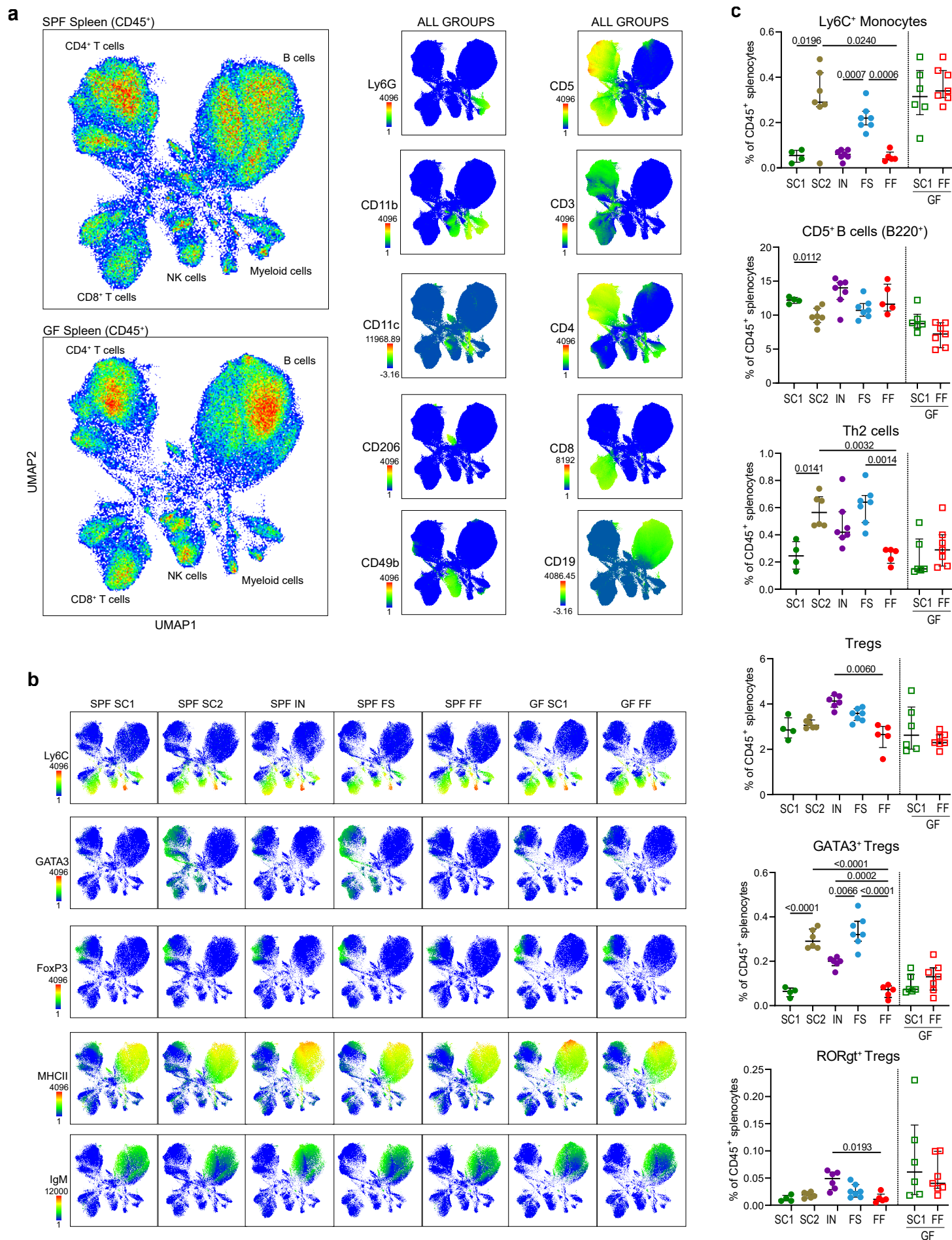

FigS3

**a**

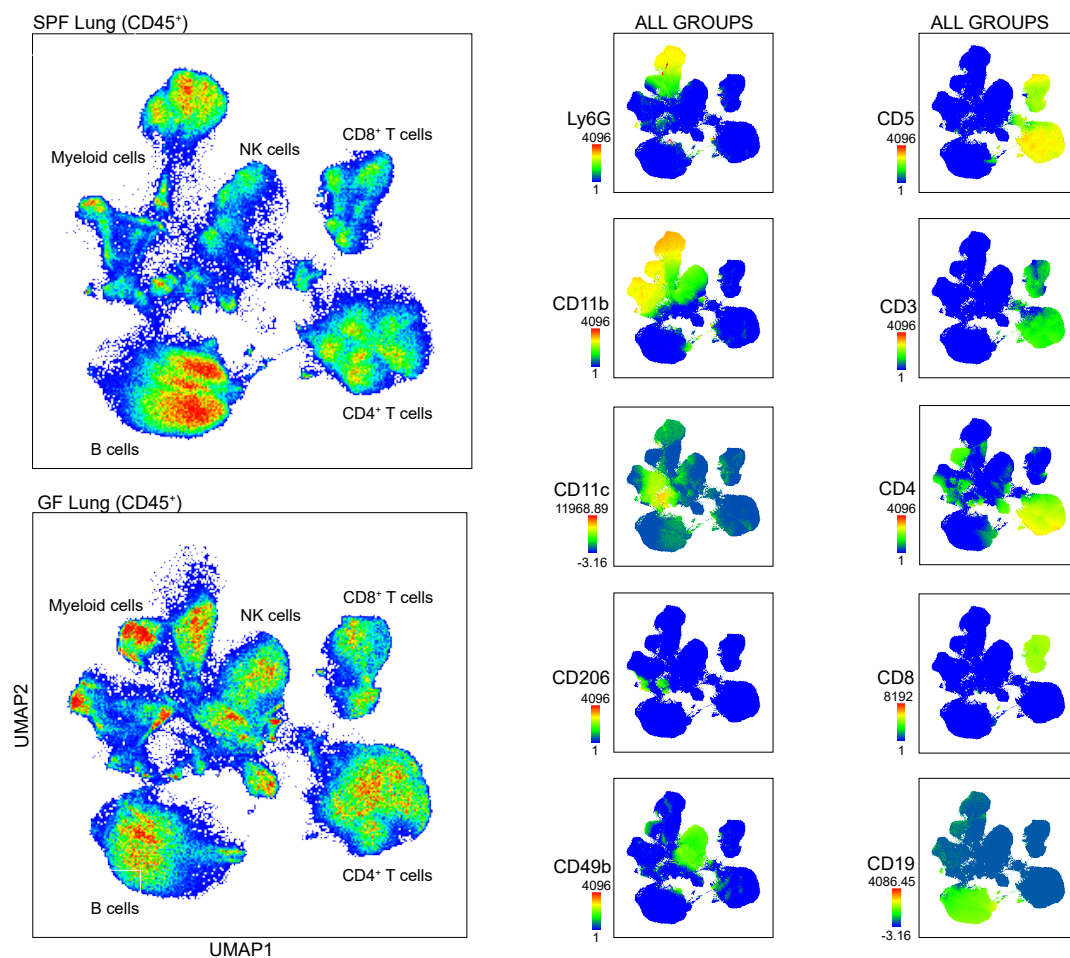

**b**

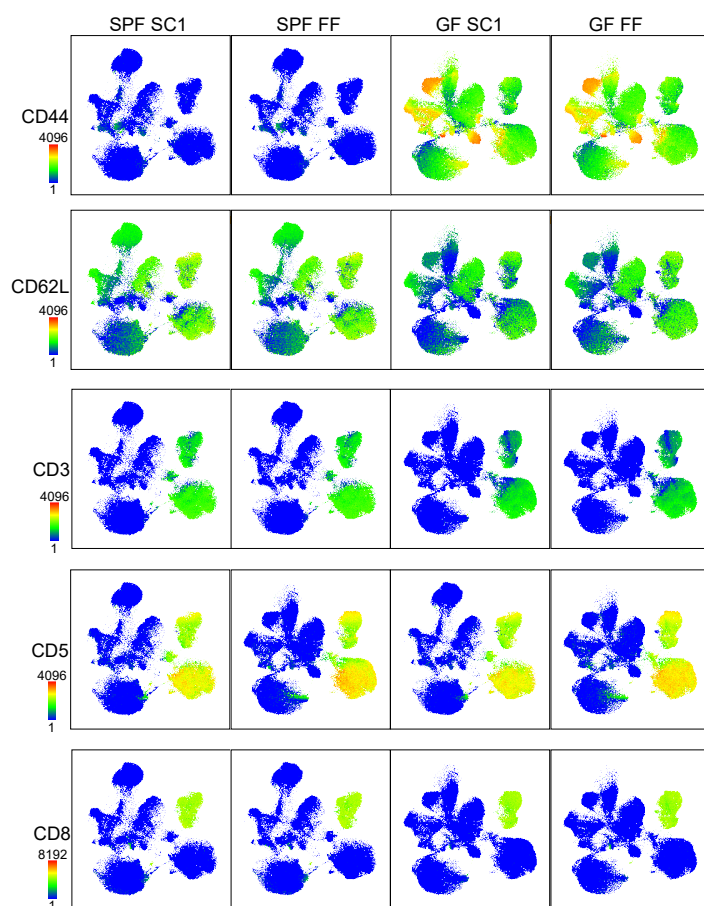

**c**

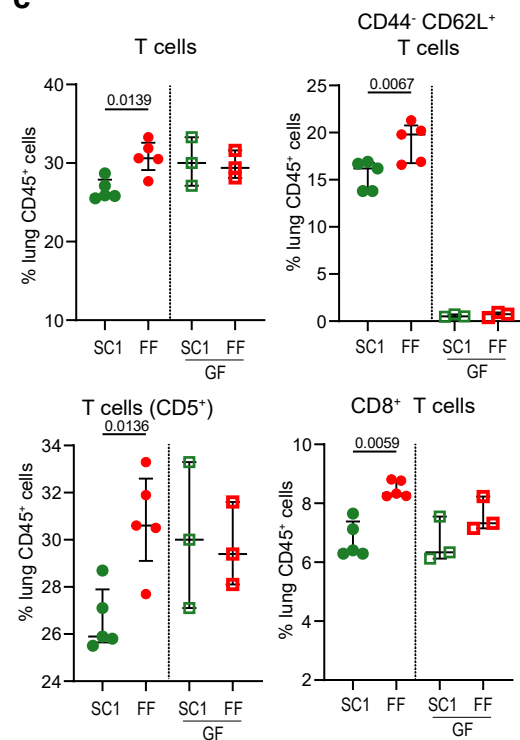

FigS4

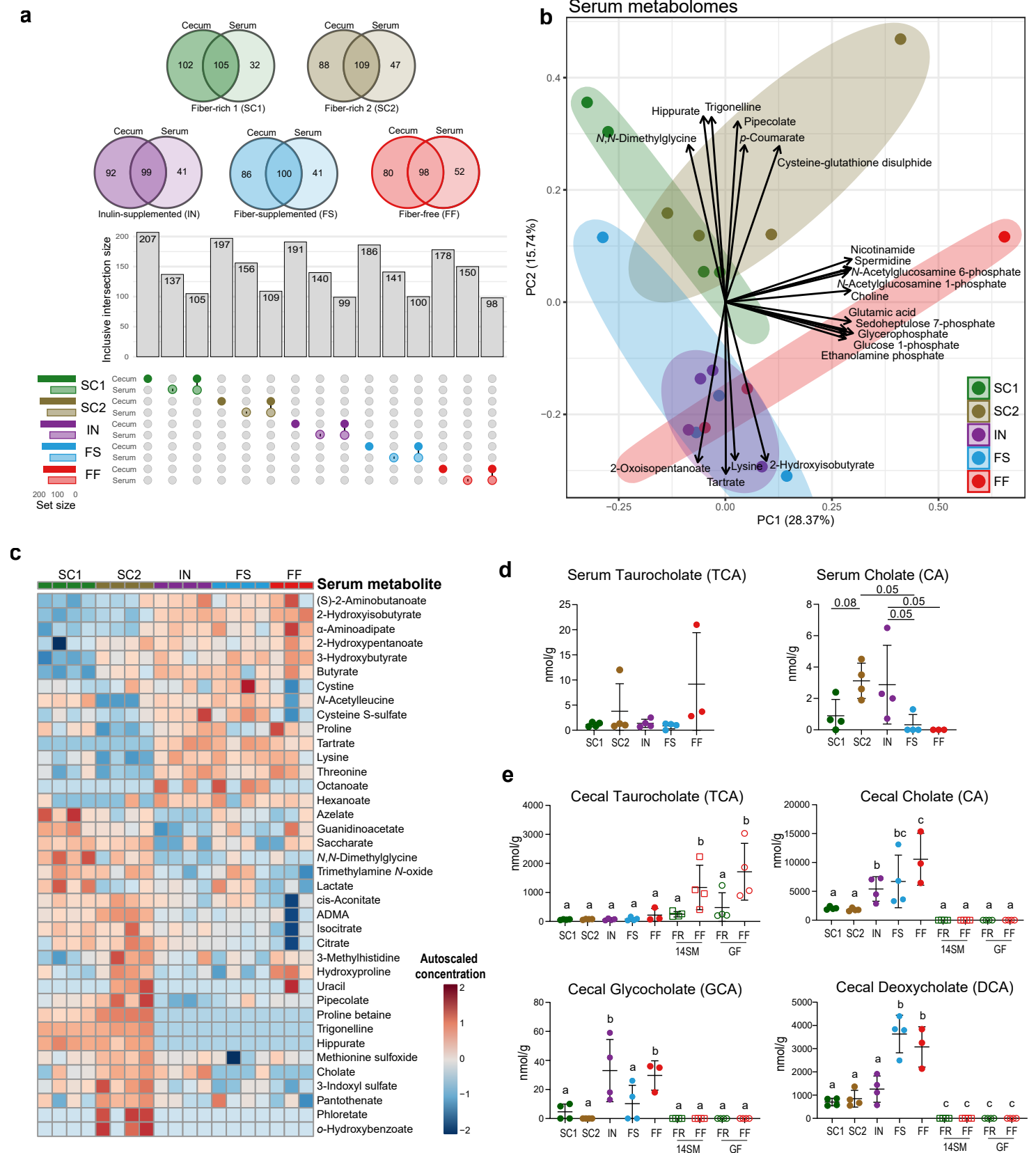

**a**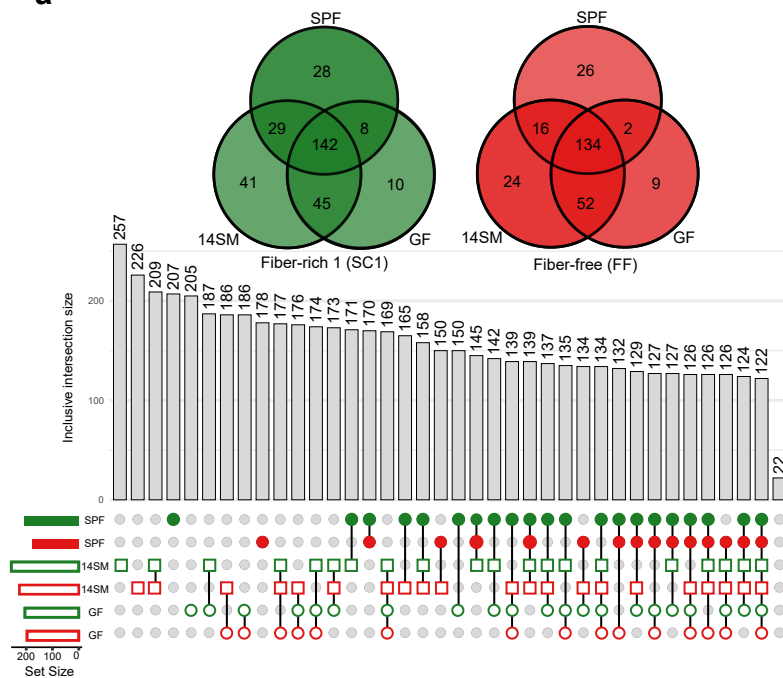**b**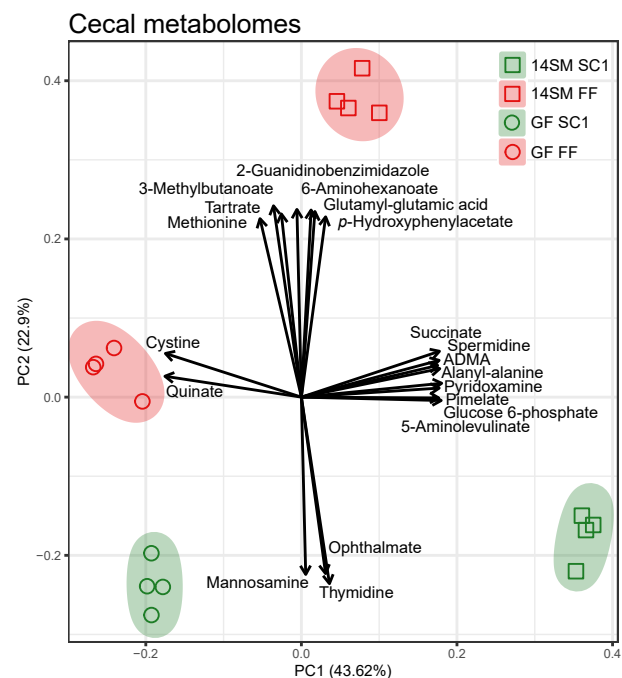**c**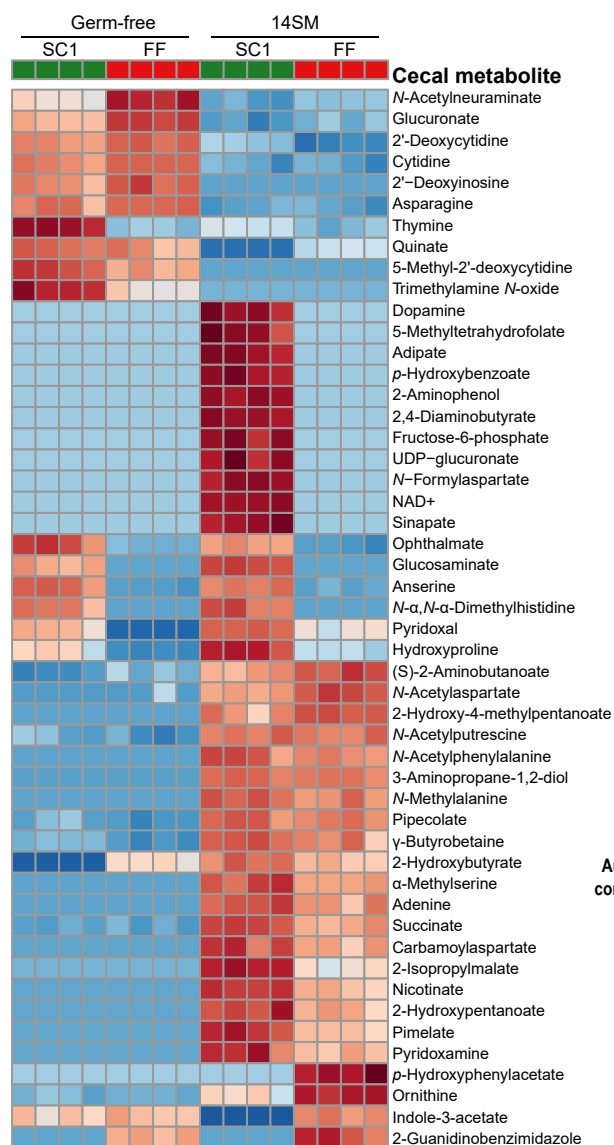

a

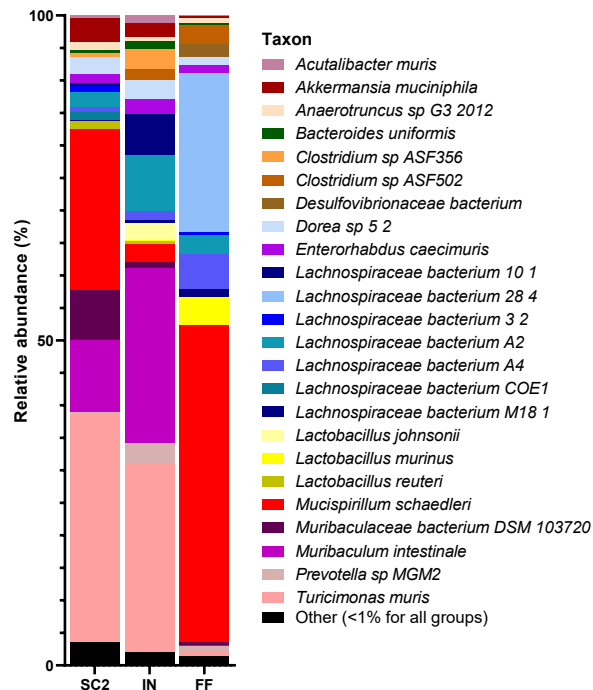

b

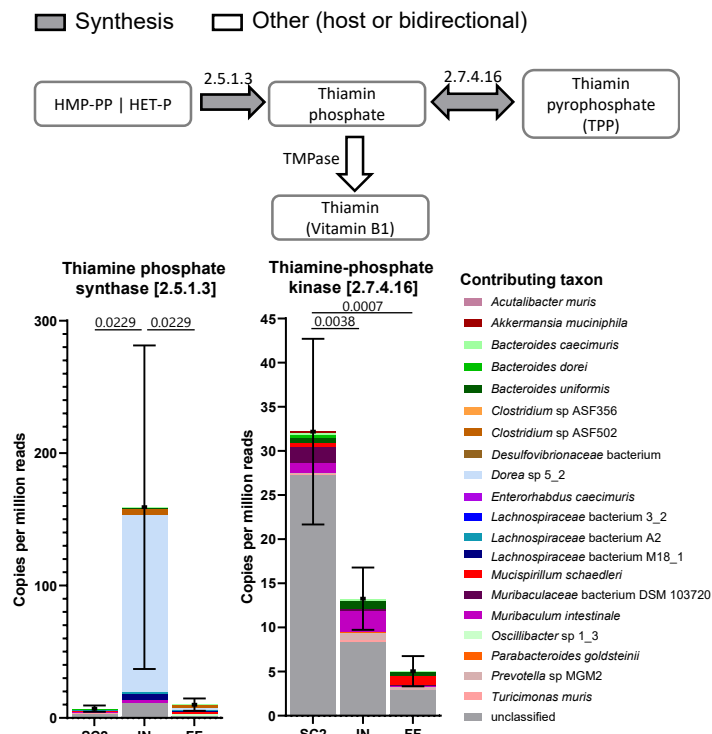

c

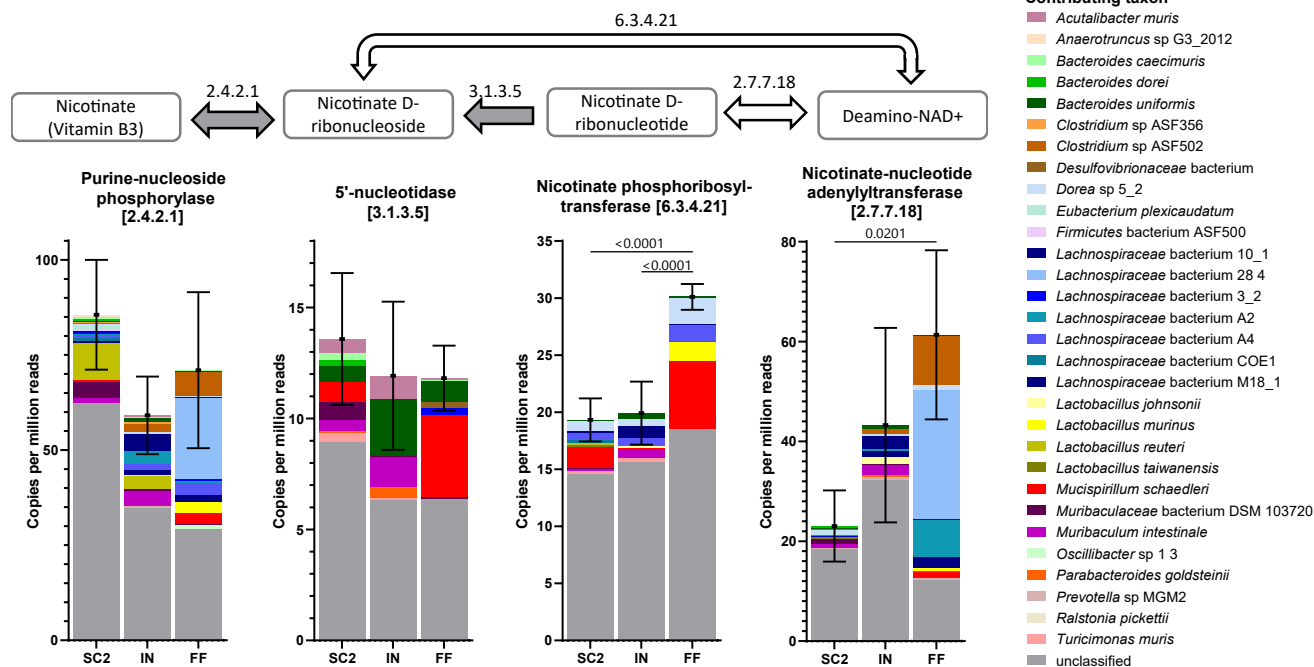

d

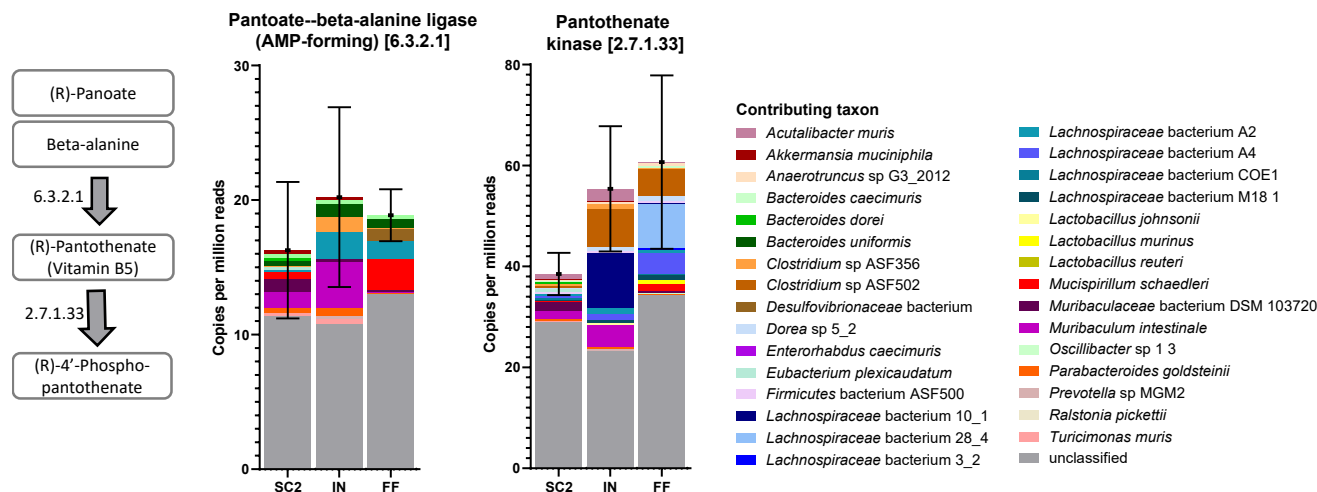

FigS7

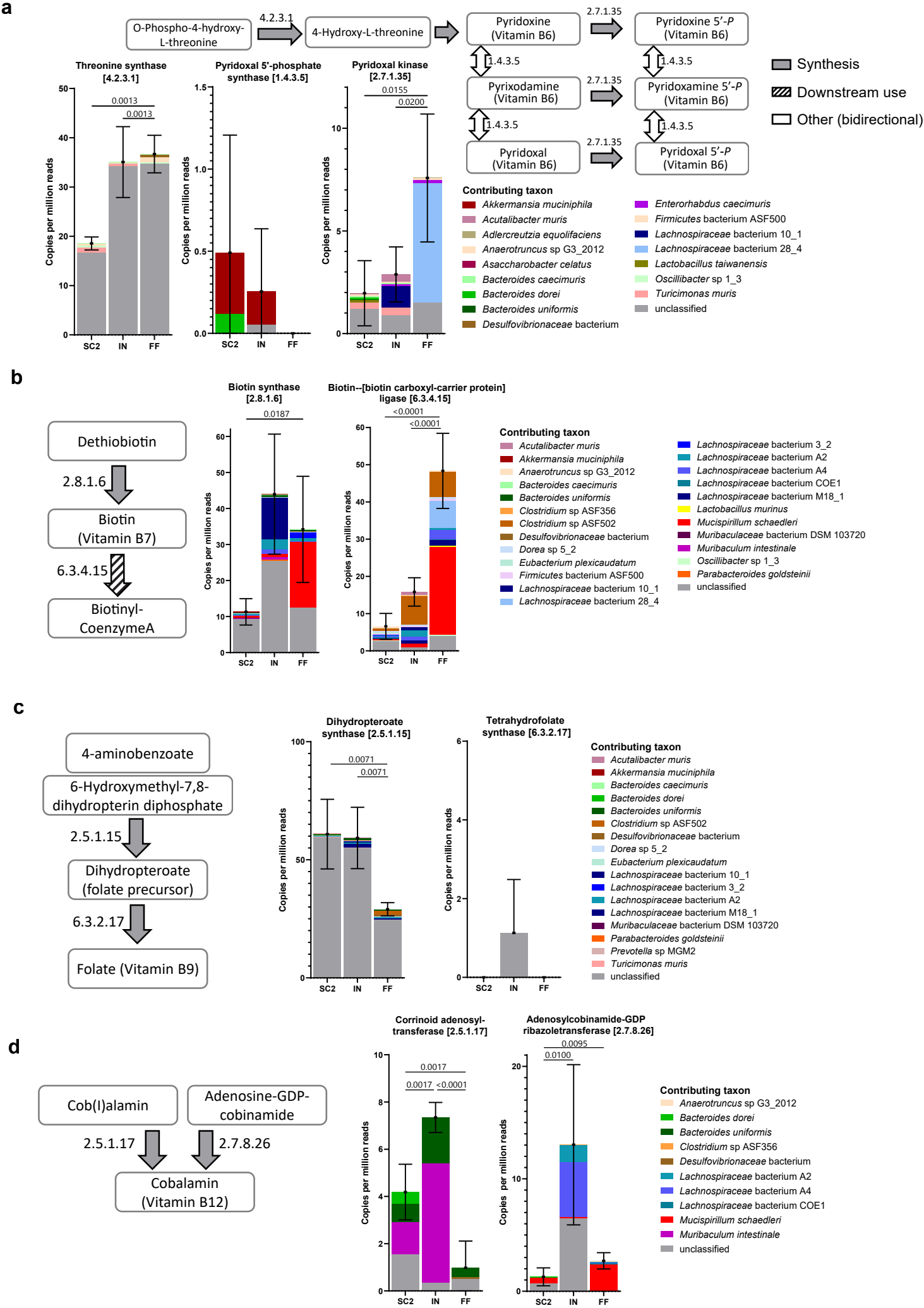

Supplement: Supplementary file 2 — Supplementary Material 1: Supplementary Figure 1. Annotated cluster heatmap of colonic lamina propria (cLP) analysis. CD45+ single live cells were imported into R for FlowSOM analysis. Based on the expression of 26 markers columns), 100 clusters were generated and manually merged based on marker expression (columns) to form 30 biologically relevant cell populations (rows). Percentages in parentheses next to the population name represent the percentage of that population among CD45+ cells. Marker expression is normalized between 0 and 1. Supplementary Figure 2. CyTOF analysis reveals systemic immune changes from a fiber-free diet. a) UMAP visualization of CD45+ immune cells isolated from the spleens of SPF (top) and GF (bottom) mice, alongside the markers used to elucidate main cell subsets (all groups pooled). c) Select marker expression facetted by colonization state and diet. Samples within each group were downsampled and then concatenated such that 60,000 events were analyzed per group (420,000 total). c) Splenic immune cell populations significantly different between specific-pathogen-free (SPF) mice fed a standard chow 1 (SC1), standard chow 2 (SC2), inulin-supplemented (IN), fiber-supplemented (FS) or fiber-free (FF) diet, alongside germ-free (GF) mice fed an SC1 or FF diet, represented as % of total CD45+ cells. Statistical testing was performed using Brown-Forsythe and Welch ANOVA or Kruskal-Wallis test with P values adjusted using the Benjamini–Hochberg method. n=4 (SPF SC1), 8 (SC2, IN, FS; 2 batches of 3–4 mice), 5 (SPF FF), or 6–7 (GF SC1, GF FF; 2 batches of 3–4 mice) mice per group. Only populations with significant differences between the diets are shown; see Supplementary Table 4 for complete list of populations analyzed. For SC2 and FS, one sample per diet was not analyzed by CyTOF due to low viable cell count; for IN, one sample (In4) was excluded from subsequent analyses due to low event count. Supplementary Figure 3. CyTOF analysis reveals pulmon [file 40168_2024_1898_MOESM1_ESM.pdf]
